# Outcomes for patients with alcohol‐related liver disease admitted to Scottish intensive care units 2010–2018

**DOI:** 10.1111/anae.16599

**Published:** 2025-03-11

**Authors:** Alaina Shariff, Stella Rhode, Stuart J. Forbes, Nazir Lone, Annemarie Docherty

**Affiliations:** ^1^ College of Medicine and Veterinary Medicine University of Edinburgh Edinburgh UK; ^2^ Usher Institute for Population Health Sciences and Informatics University of Edinburgh Edinburgh UK; ^3^ Centre for Regenerative Medicine, Institute for Regeneration and Repair University of Edinburgh Edinburgh UK; ^4^ Department of Anaesthesia Critical Care, and Pain Medicine, School of Clinical Sciences, University of Edinburgh Edinburgh UK

**Keywords:** alcohol‐related liver disease, ICU mortality, ICU re‐admission, organ support

## Abstract

**Introduction:**

Alcohol‐related liver disease is recognised as a major cause of liver‐related morbidity and mortality. Our aim was to report the prevalence of, and outcomes from, alcohol‐related liver disease after admission to ICUs in Scotland.

**Methods:**

We performed a secondary data analysis using linked Scottish national databases of all adult general ICUs in Scotland. We compared emergency non‐surgical patients admitted to ICU with and without alcohol‐related liver disease. The primary outcome was ICU mortality, and secondary outcomes were ICU admission rate ratio; 60‐day mortality; 2‐year mortality; duration of ICU and hospital stay; and need for hospital readmission.

**Results:**

Of the 49,420/103,103 (47.9%) patients admitted to ICU with emergency non‐surgical diagnoses between 2010 and 2018, we identified 2629/49,420 (5.3%) patients with alcohol‐related liver disease. Patients with alcohol‐related liver disease were more likely to receive three‐organ support (14.0% vs. 10.0%, p < 0.001). Mortality in the ICU was higher in patients with alcohol‐related liver disease (964/2629 (36.7%) vs. 10,517/46,791 (22.5%), respectively; aOR 2.03 (95%CI 1.85–2.24)). Patients with alcohol‐related liver disease who specifically presented to ICU with a gastrointestinal bleed had a lower ICU mortality (95/487 (19.5%)). Sixty‐day mortality of patients with alcohol‐related liver disease increased with higher levels of organ support (186/516 (36.0%) mortality with zero organs supported vs. 162/196 (82.7%) mortality with three organs supported).

**Discussion:**

Early mortality was high in patients with alcohol‐related liver disease who were admitted to ICU, especially if multi‐organ support was required. However, nearly one‐fifth of patients on multi‐organ support survived to hospital discharge. Early mortality for patients with alcohol‐related liver disease admitted with a gastrointestinal bleed was considerably lower and should be taken into consideration when considering management in ICU. In discussion with the patient and hepatologists, a trial of organ support with continuous re‐evaluation may be appropriate.

## Introduction

Alcohol‐related liver disease (ALD) is a major cause of liver‐related morbidity and mortality [1]. In Scotland, 80.7% of chronic liver disease deaths were attributed to ALD in 2022, and patients with ALD continue to have a higher mortality rate compared with other liver disease (14 deaths per 100,000 population compared with 3.4 per 100,000 in 2022) [2]. In the past 20 years, the number of premature deaths from ALD in England has increased by 74.2% [3]. Trends in ALD‐related hospitalisations have been relatively static over the past decade in Scotland; ALD accounted for 122 age‐standardised hospital stays per 100,000 population in 2022/2023 compared with 124 hospital stays per 100,000 in 2012/2013 [4]. Despite this, the prevalence of ALD in the community continues to rise, especially in areas of deprivation with higher alcohol consumption [1, 5].

Patients with ALD are vulnerable to recurrent, acute‐on‐chronic events such as gastrointestinal variceal bleeds and renal failure, and may require treatment in ICU [6, 7]. Early outcomes after critical care admission for patients with ALD are poor, with a reported ICU mortality between 34% and 63% [8, 9, 10], increasing to 96.7% if three‐organ support is required [11, 12]. However, very few studies have reported long‐term outcomes for patients with ALD and critical illness [8, 10, 13] with the largest national cohort study now nearly 14 years old [10]. Lastly, the longitudinal prevalence of ALD in ICU has not been previously reported.

We performed a retrospective, national cohort study using linked Scottish databases. Specifically, we aimed to identify trends in ICU admission, organ support and short and long‐term outcomes for patients with ALD. This work is important because there is a significant physiological and psychological burden associated with ICU admission and survivorship against which benefits of ICU treatments should be balanced [14]. By understanding the short‐ and long‐term consequences of an ICU stay for patients with ALD, we can better inform the shared decision‐making process, involving the patient and their families prior to ICU admission.

## Methods

Approval for the study was obtained from the Public Benefit and Privacy Panel for Health and Social Care. An ethics waiver was granted by the Usher Institute Research Ethics Committee, University of Edinburgh. All data were anonymised before release.

We performed a secondary data analysis using the following linked national databases in Scotland: Scottish Intensive Care Society Audit Group database for all adult general intensive care activity; Scottish Morbidity Record of acute hospital admissions (SMR01); and National Records Scotland for all deaths registered in Scotland. These databases demonstrate high coding accuracy and maintain a high standard of data quality through quality assurance processes at the point of data entry and through case‐note validation [15].

The cohort included all patients aged ≥18 years old admitted to level 3 and combined level 2 and 3 general ICUs with emergency non‐surgical diagnoses in Scotland from 1 January 2010 to 31 December 2018 (online Supporting Information Table S1). We compared patients with and without ALD. We defined ALD using ICD‐10 diagnostic codes in any preceding hospital admission for alcoholic fatty liver; alcoholic hepatitis; alcoholic fibrosis and sclerosis of the liver; alcoholic cirrhosis of liver; alcoholic hepatic failure; and/or unspecified alcoholic liver disease (online Supporting Information Table S2).

We included the following variables: age (categorised as per the APACHE 2 score [16]); sex; and socio‐economic status (Scottish Index of Multiple Deprivation (SIMD) quintile) [17]. Pre‐admission factors included APACHE diagnosis at ICU admission; health board (regional authority) of admitting hospital; year of admission; Charlson Comorbidity Index [18]; hospital admissions related to decompensated liver failure in the 2 years before index admission (ascites, hepatic encephalopathy and gastrointestinal variceal bleed, online Supporting Information Table S3); admission illness severity including Acute Physiology Score (physiology component of APACHE 2 score); APACHE 2 score; bilirubin levels; and level of organ support received on day one.

Our primary outcome was ICU mortality. Secondary outcomes were ICU admission rate ratio; 72 h mortality; hospital mortality; 60‐day mortality; 2‐year mortality; 5‐year mortality; ICU and hospital duration of stay; 2‐year ICU readmission; and 2‐year hospital readmission. Patients were followed up to 31 December 2018 or until death, if sooner. We hypothesised that patients with ALD who had been previously admitted to hospital with evidence of acute hepatic decompensation would have poorer outcomes compared with patients with ALD without a previous admission for hepatic decompensation. Consistent with European Association for the Study of the Liver guidelines, we used SMR01 ICD‐10 codes to identify a subgroup of those with a hospital admission related to decompensated liver disease (hepatic encephalopathy, ascites or gastrointestinal variceal bleed) [19] in the presence of ALD in the 2 years before index admission (online Supporting Information Table S3). We compared baseline characteristics, presenting diagnosis and ICU outcomes, particularly organ support requirement, mortality and 2‐year ICU readmission rate. Patients with ALD admitted to ICU with a gastrointestinal variceal bleed are a distinct cohort compared with the rest of the ALD cohort and have been known to have better short‐term outcomes [13]. We performed a subgroup analysis on this cohort, comparing baseline characteristics and outcomes with non‐variceal bleed patients within the ALD cohort. To explore whether patients with ALD had different outcomes from patients with non‐ALD liver disease, we performed additional logistic regression analyses for ICU mortality: first, with ALD vs. non‐ALD as the explanatory variable in a cohort restricted to patients with liver disease; and second with ALD vs. no liver disease in the general cohort.

Data were accessed through the Scottish National Safe Haven and analysed using R (v3.6.4, R Computing, Vienna, Austria). We were unable to report the range for median values due to the disclosure risk associated with requesting data values < 10 from the National Safe Haven. For univariable comparisons of continuous data, the Mann–Whitney U or Kruskal–Wallis test was used. For categorical comparisons, χ^2^ tests were used. Poisson regression was performed to calculate admission rate ratios for ICU admission rates. Kaplan–Meier curves reported 60‐day survival from ICU admission and log‐rank test was used for significance testing. We used cumulative incidence to assess readmission frequency (Fine‐Gray's test).

We undertook multivariable logistic regression to account for confounders in the association between ALD status and ICU mortality. We adjusted for the following variables: age; sex; SIMD; total number of Charlson comorbidities (excluding liver disease); APACHE ICU admission diagnosis by system; Acute Physiology Score; and organ support on day one of ICU admission. Missing data were addressed through a complete case analysis.

## Results

From 1 January 2010 to 31 December 2018, 103,103 patients had an index admission to a general ICU in Scotland, of whom 49,420 (47.9%) were adults admitted with emergency non‐surgical diagnoses. Of these patients, 2629 (5.3%) had a comorbid diagnosis of ALD (online Supporting Information Figure S1). There were differences in ICU admissions between health boards, and over time between health boards, but overall, there was a small but significant fall in ICU admissions across all Scottish health boards from 6.2 to 5.1 per 100,000 population across all years (admission rate ratio (ARR) 0.98, 95%CI 0.96–0.99; online Supporting Information Figure S2, Tables S4 and S5).

Patients with ALD were younger compared with patients without ALD (median (IQR) 54 y (46–61) vs. 59 y (44–71)); more likely to be male (1666/2629 (63.4%) vs. 26,392/46,553 (56.7%)); to live in deprived areas (SIMD1 997/2607 (38.2%) vs. 13,249/45,849 (28.9%)); and have fewer extra‐hepatic comorbidities (≥ 2 comorbidities excluding liver disease 562/2629 (21.4%) vs. 13,867/46,553 (29.8%)) (Table 1). Patients with ALD had higher illness severity scores (median (IQR) Acute Physiology Score 18 (8–20) vs. 14 (8–20); median (IQR) APACHE 2 score 23 (16–29) vs. 18 (11–24)) and higher bilirubin levels (55 (21–113) μmol.l^‐1^ vs. 11 (6–19) μmol.l^‐1^) on admission compared with patients without ALD (Table 1). In patients with ALD, the most frequent admission diagnosis by organ system was gastrointestinal disorders (910/2620, 34.7%). Gastrointestinal bleeding was the most common diagnosis (487/2629, 18.5%) followed by other gastrointestinal disorders (421/2629, 16%) and respiratory infection (314/2629, 11.9%) (Table 1).

**Table 1:** Baseline characteristics of patients with and without alcohol‐related liver disease (ALD) admitted with emergency non‐surgical diagnoses to general ICUs in Scotland from 1 January 2010 to 31 December 2018 (n = 49,420). Values are number (proportion) or median (IQR). Range is not reported with median (IQR) due to disclosure risk for values < 10.

Variable	Non‐ALD	ALD	Total
	n = 46,791	n = 2629	n = 49,420
Age; y	59 (44–71)	54 (46–61)	59.0 (44–70)
Sex; male	26,392 (56.7%)	1666 (63.4%)	28,058 (57.0%)
Missing	*	*	238
Scottish Index of Multiple Deprivation			
1 (most deprived)	13,249 (28.9%)	997 (38.2%)	14,246 (29.4%)
2	11,359 (24.8%)	656 (25.2%)	12,015 (24.8%)
3	8777 (19.1%)	419 (16.1%)	9196 (19.0%)
4	6881 (15.0%)	310 (11.9%)	7191 (14.8%)
5 (least deprived)	5583 (12.2%)	225 (8.6%)	5808 (12.0%)
Missing	942	22	964
Total Charlson comorbidities**			
0	17,531 (37.7%)	1205 (45.8%)	18,736 (38.1%)
1	15,155 (32.6%)	862 (32.8%)	16,017 (32.6%)
≥ 2	13,867 (29.8%)	562 (21.4%)	14,429 (29.3%)
Missing	*	*	238
APACHE ICU admission diagnosis (system)			
Gastrointestinal disorder	3901 (8.4%)	910 (34.7%)	4811 (9.8%)
Cardiovascular disorder	14,270 (30.6%)	529 (20.2%)	14,799 (30.1%)
Respiratory disorder	11,916 (25.6%)	535 (20.4%)	12,451 (25.3%)
Neurological disorder	7238 (15.5%)	386 (14.7%)	7624 (15.5%)
Metabolic/renal disorder	6125 (13.2%)	198 (7.6%)	6323 (12.9%)
Trauma	3125 (6.7%)	62 (2.4%)	3187 (6.5%)
Missing	*	*	225
APACHE ICU admission diagnosis^#^			
Respiratory infection	6200 (13.3%)	314 (11.9%)	6514 (13.2%)
Sepsis	4809 (10.3%)	299 (11.4%)	5108 (10.3%)
Other gastrointestinal disorder^†^	3251 (6.9%)	421 (16.0%)	3672 (7.4%)
ICH/SDH/SAH	1909 (4.1%)	170 (6.5%)	2079 (4.2%)
Gastrointestinal bleeding	604 (1.3%)	487 (18.5%)	1091 (2.2%)
Other	30,018 (64.2%)	938 (35.7%)	30,956 (62.6%)
Acute Physiology Score	14 (8–20)	18 (11–24)	14 (8–20)
APACHE 2 score	18 (11–24)	23 (16–29)	18.0 (11–25)
Bilirubin; μmol.l^‐1^	11 (6–19)	55 (21.0–113)	11 (6–21)

ICH, intracerebral haemorrhage; SDH, subdural haematoma; SAH, subarachnoid haemorrhage.

* Values < 10 suppressed due to disclosure risk.

** Excluding liver disease.

^#^ Five most frequent for patients with ALD. Excluding liver disease.

† Other gastrointestinal disorder comprises pancreatitis; perforation/rupture; cholangitis/cholecystitis; obstruction; hepatic failure‐toxin; vascular insufficiency/embolism/infarction; hepatic failure‐overdose; neoplasm; localised abscess/cyst; peritonitis; inflammatory disease; hepatic failure‐virus; hepatic failure‐drug reaction; diverticulosis; and acute corrosive injury.

Patients with ALD were more likely to receive all forms of organ support on day one: intermittent mandatory ventilation (1889/2626 (71.9%) vs. 28,689/46,761 (61.4%), p < 0.001); renal support (278/2626 (10.6%) vs. 3446/46,761 (7.4%), p < 0.001); and cardiovascular support (1215/2626 (46.3%) vs. 19,609/46,761 (41.9%), p < 0.001); and across their entire ICU admission: intermittent mandatory ventilation (2058/2627 (78.3%) vs. 31,252/46,773 (66.8%), p < 0.001); renal support (480/2627 (18.3%) vs. 6261/46,773 (13.4%), p < 0.001); cardiovascular support (1558/2627 (59.3%) vs. 24,527/46,773 (52.4%), p < 0.001); and three‐organ support (386/2627 (14.7%) vs. 4665/46,773 (10%), p < 0.001) compared with patients without ALD (online Supporting Information Table S6). Patients with ALD spent more total days on organ support (median (IQR) 2 (1–6) days vs. 2 (1–4) days, p < 0.001) (online Supporting Information Table S6).

**Figure 1:**
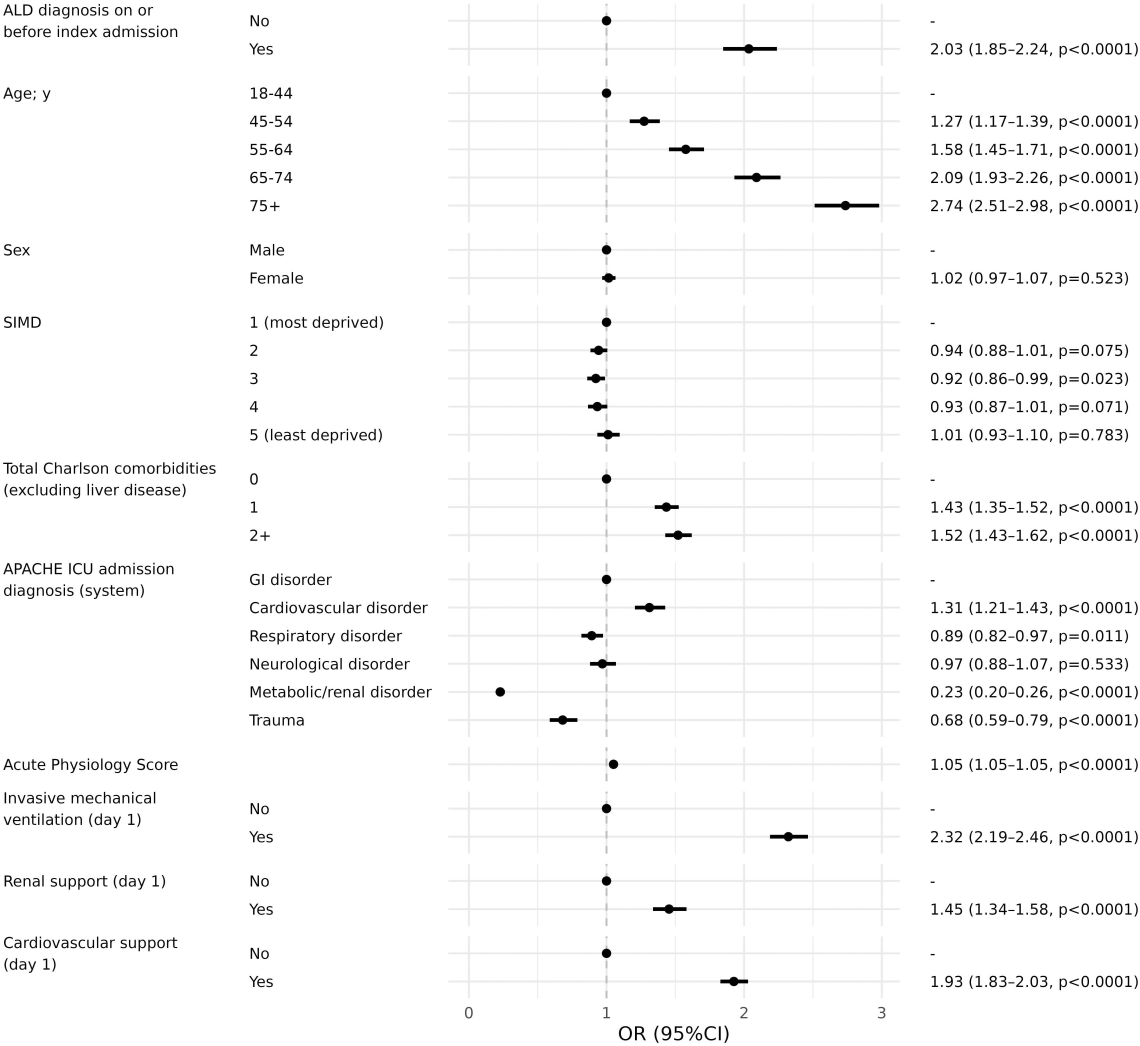
Forest ratio plot (95%CI, p value) identifying factors associated with ICU mortality in patients admitted with emergency non‐surgical diagnoses to general ICUs in Scotland from 1 January 2010 to 31 December 2018 (n = 49,420). ALD, alcohol‐related liver disease; SIMD, Scottish Index of Multiple Deprivation; GI, gastrointestinal.

**Figure 2:**
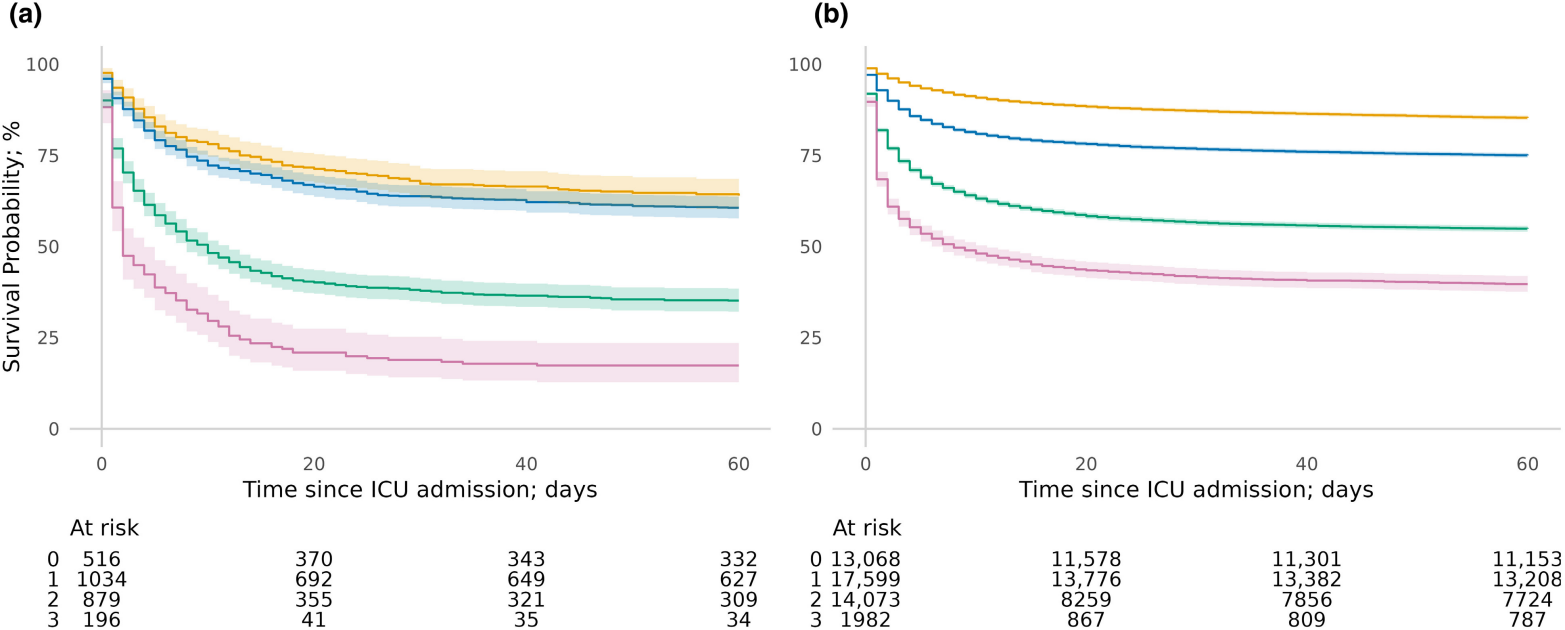
Kaplan–Meier survival analysis plot of the probability of survival to 60‐days from ICU admission for (a) ALD cohort (n = 2629) and (b) patients without ALD cohort (n = 46,791) from 1 January 2010 to 31 December 2018, stratified by level of organ support use in ICU. Yellow, zero organs supported; blue, one organ supported; green, two organs supported; purple, three organs supported.

In patients who died on ICU, duration of stay in ICU was similar in those with and without ALD (median (IQR) 2 (1–5) days vs. 2 (1–5) days, respectively; p = 0.101) (Table 3). In patients who survived to ICU discharge, those with ALD had a longer duration of stay in ICU (median (IQR) 3 (1–7) days vs. 3 (1–6) days, p < 0.001) (Table 3). Overall, early mortality was high in patients with ALD, with 636/2628 (24.2%) dying in the first 72 h (patients without ALD, 7409/46,752 (15.8%), p < 0.001) and 964/2628 (36.7%) dying in ICU (patients without ALD, 10,517/46,739 (22.5%), p < 0.001) (Table 2, online Supporting Information Figure S3). After controlling for confounding factors, patients with ALD experienced higher ICU mortality compared with patients without ALD (aOR 2.03, 95%CI 1.85–2.24, p < 0.001) (Fig. 1 and online Supporting Information Table S8). Mortality increased with higher level of organ support required in both cohorts; however, patients with ALD had a considerably higher mortality with multi‐organ support. Patients with ALD who received three‐organ support had a mortality of 162/196 (82.7%) at 60‐days compared with 1195/1982 (60.3%) in patients without ALD (online Supporting Information Table S7 and Fig. 2).

**Table 2:** Mortality rates for patients with and without alcohol‐related liver disease (ALD) admitted with emergency non‐surgical diagnoses to general ICUs in Scotland from 1 January 2010 to 31 December 2018. Hospital and ICU duration of stay is reported for all patients and is also stratified by survival status. Values are number (proportion).

Outcome	Non‐ALD	ALD	Total	p value
	n = 46,791	n = 2629	n = 49,420	
72‐h mortality	7409 (15.8%)	636 (24.2%)	8045 (16.3%)	< 0.0001
Missing	*	*	40	
ICU mortality	10,517 (22.5%)	964 (36.7%)	11,481 (23.3%)	< 0.0001
Missing	*	*	53	
Hospital mortality	13,366 (29.4%)	1297 (49.9%)	14,663 (30.5%)	< 0.0001
Missing	1312	31	1343	
60‐day mortality	13,883 (29.7%)	1326 (50.5%)	15,209 (30.8%)	< 0.0001
Missing	*	*	40	
2‐year mortality	18,342 (43.9%)	1693 (68.1%)	20,035 (45.2%)	< 0.0001
Missing	4979	144	5123	
5‐year mortality	21,227 (64.3%)	1926 (86.0%)	23,153 (65.6%)	< 0.0001
Missing	13,761	390	14,151	

* Values < 10 suppressed due to disclosure risk.

Duration of hospital stay was longer for patients with ALD for both hospital survivors (median (IQR) 16 (8–31) days vs. 12 (IQR 5–25) days, p < 0.001) and hospital non‐survivors (median (IQR) 4 (1–10) days vs. 3 (1–8) days, p < 0.001) (Table 3). A total of 1297/2598 (49.9%) patients with ALD died in hospital (patients without ALD 13,366/45,479 (29.4%), p < 0.001) (Table 2 and online Supporting Information Figure S3).

**Table 3:** Duration of stay in ICU and hospital for patients with and without alcohol‐related liver disease (ALD) admitted with emergency non‐surgical diagnoses to general ICUs in Scotland from 1 January 2010 to 31 December 2018. Outcomes are stratified based on ICU and hospital survivorship. Values are median (IQR). Range is not reported with median (IQR) due to disclosure risk for values < 10.

Variable	Non‐ALD	ALD	Total	p value
	n = 46,791	n = 2629	n = 49,420	
ICU duration of stay; d	2 (1–6)	3 (1–6)	2 (1–6)	0.0330
Hospital duration of stay post ICU admission; d	9 (3–21)	9.0 (3–21)	9 (3–21)	0.8016

	n = 36,222	n = 1664	n = 37,886	
ICU duration of stay (ICU survivors); d	3 (1–6)	3 (1–7)	3 (1–6)	0.0003

	n = 10,517	n = 964	n = 11,481	
ICU duration of stay (ICU non‐survivors); d	2 (1–5)	2 (1–5)	2 (1–5)	0.1010

	n = 32,113	n = 1301	n = 33,414	
Hospital duration of stay (survivors); d	12 (5–25)	16 (8–31)	12 (5–26)	< 0.0001

	n = 13,366	n = 1297	n = 14,663	
Hospital duration of stay (hospital non‐survivors); d	3 (1–8)	4 (1–10)	3 (1–9)	0.0001

In patients with ALD who survived to hospital discharge, mortality at 2 years was 1693/2485 (68.1%) compared with 18,342/41,812 (43.9%) patients without ALD (p < 0.001). At 5 years, mortality was 1926/2239 (86.0%) in patients with ALD compared with 21,227/33,030 (64.3%) in those without ALD (p < 0.001) (Table 2 and online Supporting Information Figure S3).

Patients with ALD were more likely to be readmitted to ICU (253/1167 (21.7%) vs. 2554/27,439 (9.3%), p < 0.001) and to hospital (1086/1265 (85.8%) vs. 19,975/29,781 (67.1%), p < 0.001) within the next 2 years (Table 4). The cumulative incidence of hospital readmission reached 657/1280 (51%, 95%CI 48–54%) within 3 months and 998/1255 (80%, 95%CI 76–81%) within 1 year for those patients with ALD (online Supporting Information Figure S4).

**Table 4:** Two‐year readmission to ICU and hospital for all hospital survivors admitted with emergency non‐surgical diagnoses to general ICUs in Scotland from 1 January 2010 to 31 December 2018. Values are number (proportion).

Outcome	Non‐ALD	ALD	Total	p value
	n = 32,113	n = 1301	n = 33,414	
ICU readmission within 2 years	2554 (9.3%)	253 (21.7%)	2807 (9.8%)	< 0.0001
Missing	4674	134	4808	
Hospital readmission within 2 years	19,975 (67.1%)	1086 (85.8%)	21,061 (67.8%)	< 0.0001
Missing	2332	36	2368	

Patients with previously decompensated ALD were younger (median (IQR) 52 y (43–58) vs. 54 y (46–62)) and more comorbid (≥ 2 comorbidities, 189/787 (24%) vs. 373/1842 (20.2%)) compared with patients without previously decompensated ALD (online Supporting Information Table S9), but there was no significant difference in illness severity (median (IQR) Acute Physiology Score 17 (11–23) vs. 18 (11–24), p = 0.091). Gastrointestinal variceal bleed was the most common ICU admission diagnosis in patients with previously decompensated ALD (211/787 (26.8%)) (online Supporting Information Table S9). Patients with previously decompensated ALD spent fewer total days on organ support (median (IQR) 2 (1–4) days vs. 2 (1–6) days, p = 0.005) and required less maximum organ support (98/787 (12.5%) vs. 288/1840 (15.7%), p = 0.007) (online Supporting Information Table S10).

Patients with previously decompensated ALD had a lower ICU mortality (257/786 (32.7%) vs. 707/1842 (38.4%), p = 0.006) (online Supporting Information Tables S10 and S11), but had a higher 2‐year mortality (541/756 (71.6%) vs. 1152/1729 (66.6%), p = 0.017) (online Supporting Information Figure S5 and Table S11). In patients who died on ICU, there was no significant difference in duration of ICU stay (median (IQR) 2 (1–4) days vs. 2 (1–6) days, p = 0.067) or hospital stay (median (IQR) 3.5 (1–10) days vs. 4 (1–10) days, p = 0.899) between those with and without ALD (online Supporting Information Table S12). Among survivors, patients with previously decompensated ALD had a shorter duration of ICU stay (median (IQR) 3 (1–6) days vs. 3 (1–8) days, p = 0.010) and in hospital stay (median (IQR) 14 (7–29) days vs. 17 (8–32) days, p = 0.002) (online Supporting Information Table S12). Patients with previously decompensated ALD were more likely to be readmitted to ICU within 2 years following their index ICU admission (108/376 (28.7%) vs. 145/791 (18.3%), p < 0.001) (online Supporting Information Table S13).

Within patients with ALD, those admitted to ICU with a gastrointestinal variceal bleed had fewer comorbidities (≥ 2 comorbidities 87/487 (17.9%) vs. 474/2133 (22.2%)) and lower illness severity scores at admission (median (IQR) Acute Physiology Score 12 (8–18) vs. 19 (13–24); median (IQR) APACHE 2 19 (14–25) vs. 24 (17–30)) (online Supporting Information Table S14). These patients had lower short‐term mortality (ICU mortality 95/486 (19.5%) vs. 865/2133 (40.6%), p < 0.001) and long‐term mortality (2‐year mortality 271/455 (59.6%) vs. 1417/2021 (70.1%), p < 0.001) compared with patients with ALD but no gastrointestinal variceal bleed. There was no difference in mortality at 5 years (341/400 (85.2%) vs. 1579/1831 (86.2%), p = 0.662) (online Supporting Information Figure S6 and Table S15). There was no difference in duration of ICU stay among those patients who died on ICU (median (IQR) 3 (1–6) days vs. 2 (1–5) days, p = 0.478). Among patients who survived to ICU discharge, median (IQR) duration of ICU and hospital stay were shorter (1 (1–3) days vs. 4 (2–8) days, and 10 (6–18) days vs. 18 (9–35) days, respectively; p < 0.001 for both) (online Supporting Information Table S16). Patients with a gastrointestinal variceal bleed were more likely to be readmitted to ICU within 2 years (93/289 (32.2%) vs. 160/873 (18.3%), p < 0.001) (online Supporting Information Table S17).

Patients with ALD had increased odds of ICU mortality compared with those with non‐ALD liver disease (aOR 1.40, 95%CI 1.21–1.61, p < 0.001) (online Supporting Information Figure S7 and Table S18). When comparing patients with ALD with general medical ICU patients excluding non‐ALD liver disease, ALD was associated with increased odds of ICU mortality (aOR 2.15, 95%CI 1.95–2.37, p < 0.001) (online Supporting Information Figure S8 and Table S19).

## Discussion

Patients with ALD were younger and more unwell than patients without ALD. Early mortality was high, with one‐third of patients dying within ICU and one‐half dying within 60 days. Mortality was extremely high for patients with ALD who received multi‐organ support, although one‐fifth of these patients survived until hospital discharge. However, once patients were discharged, the mortality rate stabilised over the next 2 years. For those who survived, half were readmitted to hospital as an emergency within 3 months.

These findings are important because decisions in ICU for this cohort have historically been influenced by high early mortality rates. For selected patients with acute‐on‐chronic liver failure, aggressive treatment and organ support in ICU may provide a bridge to transplantation [20] but further work is required to identify patients most likely to benefit from this. Whilst patient characteristics at admission can guide these decisions, ultimately it should involve a careful discussion with the patient, their family and other stakeholders to balance the benefits and burdens of treatment. We recommend that our data may inform these shared decisions relating to outcomes following admission to critical care in this cohort of patients.

Similar to previous studies, mortality was high for patients with ALD when compared with the general ICU population [10, 11, 13, 21, 22] and patients with non‐ALD liver disease [13], and increased with higher level of organ support [10, 11, 22]. Mortality in ICU was higher than for patients who typically present to ICU with sepsis (24.5%) [23] but similar to those who present with acute respiratory distress syndrome (36%) [24]. Mortality at 60‐days increased from 36.0% (no organ support) to 82.7% (three organ support), unchanged from a previous national Scottish cohort (2005–2010) [10]. One‐year mortality was higher in the national cohort study (66.1%) but 5‐year mortality was lower (79.2%) when compared with our data [10]. A single‐centre study found high initial mortality (35.2% in ICU) but low mortality after hospital discharge in patients with ALD cirrhosis [25].

The high mortality in our study may be influenced by the tertiary centre nature of a proportion of patients, but it is important to note that one‐fifth of the patients with ALD in our national cohort who received three‐organ support survived to hospital discharge. This is a similar proportion to patients who present after cardiac arrest [26]. The high mortality seen at 72‐h and in patients who received minimal organ support may reflect treatment escalation plans as a result of early decision‐making. Objective scoring tools such as the CLIF‐SOFA, CLIF‐C ACLF and CLIF‐C AD scores may help to guide this. For example, studies in patients who were acutely ill with cancer, a cohort similarly deemed to have poor outcome and often managed with ward level care, have shown improved survival with more intensive treatment [27, 28].

ICU mortality for patients with ALD was lower in our study (36.7%) compared with 44.1% in a similar national cohort from 2005 to 2010 [10] and 58% in a single‐centre study in 2006 [11]. Mortality among patients who received three‐organ support in our study was lower than the 91% hospital mortality reported for three‐organ support in a 2006 study [11] or the 18‐times greater risk of in‐hospital mortality as seen in a 2010 study (OR 18.0, 95%CI 5.9–69.3) [22]. Half of the patients with ALD in our cohort survived to hospital discharge, compared with one‐third in a similar study conducted in England [29]. We adjusted for illness severity at presentation to ICU, suggesting this may be due to improvements in outcome rather than differences in patient selection.

Patients with previously decompensated ALD had a relatively low mortality in ICU, which was potentially unexpected given their underlying frailty and end‐stage liver disease. However, this may be a result of the high proportion (211/787, 26.8%) of patients from the sub‐cohort presenting to ICU with gastrointestinal variceal bleeding, who often require short‐term mechanical ventilation for airway protection during therapeutic endoscopy and vasopressors for blood pressure support. Once haemostasis is achieved, subsequent requirement for organ support is short‐lived. Patients with ALD who specifically presented to ICU with a gastrointestinal bleed had a lower ICU mortality (95/486 (19.5%)), shorter stay in ICU (median (IQR) 1 (1–3) day) and were more likely to return to ICU again (93/289 (32.2%)). There was likely significant selection bias at admission to ICU given the underrepresentation of patients with previous decompensation being admitted with other diagnoses. Lastly, ICU admission rates in our study did not reflect the increasing global incidence in ALD in the community [1, 5]. This may reflect increasing patient selection before ICU admission.

Our study had several strengths. We performed a national, multicentre study of all ICU admissions in Scotland with validated, linked databases to ensure long‐term follow‐up for mortality and readmission rates. We were able to show trends in admission on a geographical scale and capture readmissions to all Scottish hospitals. Our study is one of the few to have investigated the relationship of organ support and mortality over time and report long‐term outcomes for this cohort. However, our cohort was restricted to patients admitted to ICU, and therefore does not provide evidence on patients who were potentially referred but not admitted to ICU. We defined ALD using retrospectively classified ICD‐10 diagnostic codes from combined ICU and hospital registry datasets, which may carry a risk of misclassification. We only graded the severity of illness using ICU‐specific scores. However, acute physiology‐based scoring systems may predict early mortality more accurately than traditional liver‐specific scores in patients with ALD, especially when discriminating between survivors and non‐survivors [8]; ICU‐specific scores also perform similarly to the CLIF‐SOFA liver scores in patients with liver cirrhosis [30, 31]. We were unable to perform regression analyses in our subgroup of patients with evidence of decompensation due to the profound mediating effect of diagnosis on outcomes. We were unable to differentiate outcomes between various clinical states of ALD, which may have different outcome profiles [32]. Lastly, the data were collected before the introduction of minimum unit pricing in Scotland, which may have influenced epidemiology and outcomes in critical care in more recent years [33].

Patients with a comorbid diagnosis of ALD have high early mortality rates in ICU, which increases with higher levels of organ support. There is a need for careful consideration over the escalation of organ support in patients with ALD. The decision to escalate treatment should primarily be patient‐oriented, and a trial of organ support with continuous reassessment may be appropriate, together with early involvement of hepatologists or palliative care teams as appropriate.

## Supporting information


**Figure S1.** Adult patients with index non‐surgical admission to general ICUs in Scotland stratified by ALD comorbidity.
**Figure S2.** All ICU admissions with alcohol‐related liver disease comorbidity per 100,000 population plotted over time stratified by Scottish health boards.
**Figure S3.** Kaplan–Meier survival analysis plot of the probability of survival to 5 years from ICU admission for the ALD cohort and non‐ALD cohort.
**Figure S4.** Cumulative incidence of hospital readmission (hospital survivors only) in ALD and non‐ALD cohorts.
**Figure S5.** Kaplan–Meier survival analysis plot of the probability of survival to 60‐days from ICU admission for the decompensated ALD cohort and non‐decompensated ALD cohort.
**Figure S6.** Kaplan–Meier survival analysis plot of the probability of survival to 60‐days from ICU admission for the ALD gastrointestinal bleed cohort and ALD non‐ gastrointestinal bleed cohort
**Figure S7.** Forest plot identifying factors associated with ICU mortality, restricted to only patients with a comorbid liver disease diagnosis (including ALD) admitted to ICU.
**Figure S8.** Forest plot identifying factors associated with ICU mortality in patients with ALD in ICU, excluding patients with non‐ALD liver disease.

**Table S1.** Critical care levels.
**Table S2.** ICD‐10 diagnostic codes used to define the ALD comorbidity using the SMR01 database.
**Table S3.** ICD‐10 Diagnostic codes used to define decompensated liver failure group within the ALD cohort.
**Table S4.** ICU admission rate ratios of patients with an ALD comorbidity by Scottish health boards over time
**Table S5.** All ICU admissions with an ALD comorbidity per 100,000 population over time.
**Table S6.** Type of organ support received on day 1 of ICU admission and throughout ICU admission in all patients admitted as emergency non‐surgical to Scottish ICUs.
**Table S7.** Sixty‐day mortality for ALD and non‐ALD groups, stratified by level of organ support.
**Table S8.** Results from the multivariable regression analysis identifying factors associated with ICU mortality in all patients admitted to ICU as an emergency, for non‐surgical reasons.
**Table S9.** Baseline characteristics of patients with ALD with and without a previous hospital admission related to decompensated liver failure 2 years before index ICU admission.
**Table S10.** Summary of treatment in ICU for patients with ALD with and without a previous hospital admission related to decompensated liver failure within 2 years before index ICU admission.
**Table S11.** Mortality rates for patients with ALD with and without a previous hospital admission related to decompensated liver failure in the 2 years before index ICU admission.
**Table S12.**Duration of stay in ICU and hospital for patients with ALD with and without a previous hospital admission related to decompensated liver failure in the 2 years before index ICU admission.
**Table S13.** Readmission within 2 years (hospital survivors only) following index ICU admission for patients with ALD with and without a hospital admission related to decompensated liver failure.
**Table S14.** Baseline characteristics of patients with ALD admitted to ICU with and without a gastrointestinal variceal bleed.
**Table S15.** Summary of treatment and mortality in ICU and hospital for patients with ALD admitted to ICU with and without a gastrointestinal variceal bleed.
**Table S16.** Duration of stay in ICU and hospital for patients with ALD admitted to ICU with and without a gastrointestinal variceal bleed.
**Table S17.** Readmission within 2 years (hospital survivors only) following index ICU admission for patients with ALD admitted to ICU with and without a gastrointestinal variceal bleed.
**Table S18.** Individual results for the multivariable regression analysis (Figure S5) for factors associated with ICU mortality, restricted to patients with liver disease in ICU (including ALD).
**Table S19.** Individual results for the multivariable regression analysis (Figure S6) identifying factors associated with ICU mortality in all patients in ICU excluding patients non‐ALD liver disease.
