# Supplementary material for: Outcomes for patients with alcohol‐related liver disease admitted to Scottish intensive care units 2010–2018
Source: Anaesthesia. 2025 Mar 11;80(8):904–14. doi: 10.1111/anae.16599 (PMC12256163; doi:10.1111/anae.16599)

**Figure S1** Flow diagram of adult patients with index non-surgical admission to general ICUs in Scotland 01/01/2010-31/12/2018, stratified by ALD comorbidity (n=49,420). Within the ALD population (n=2,629), patients were further stratified by evidence of hepatic decompensation within previous 2 years (Yes n=787, No n=1,842).


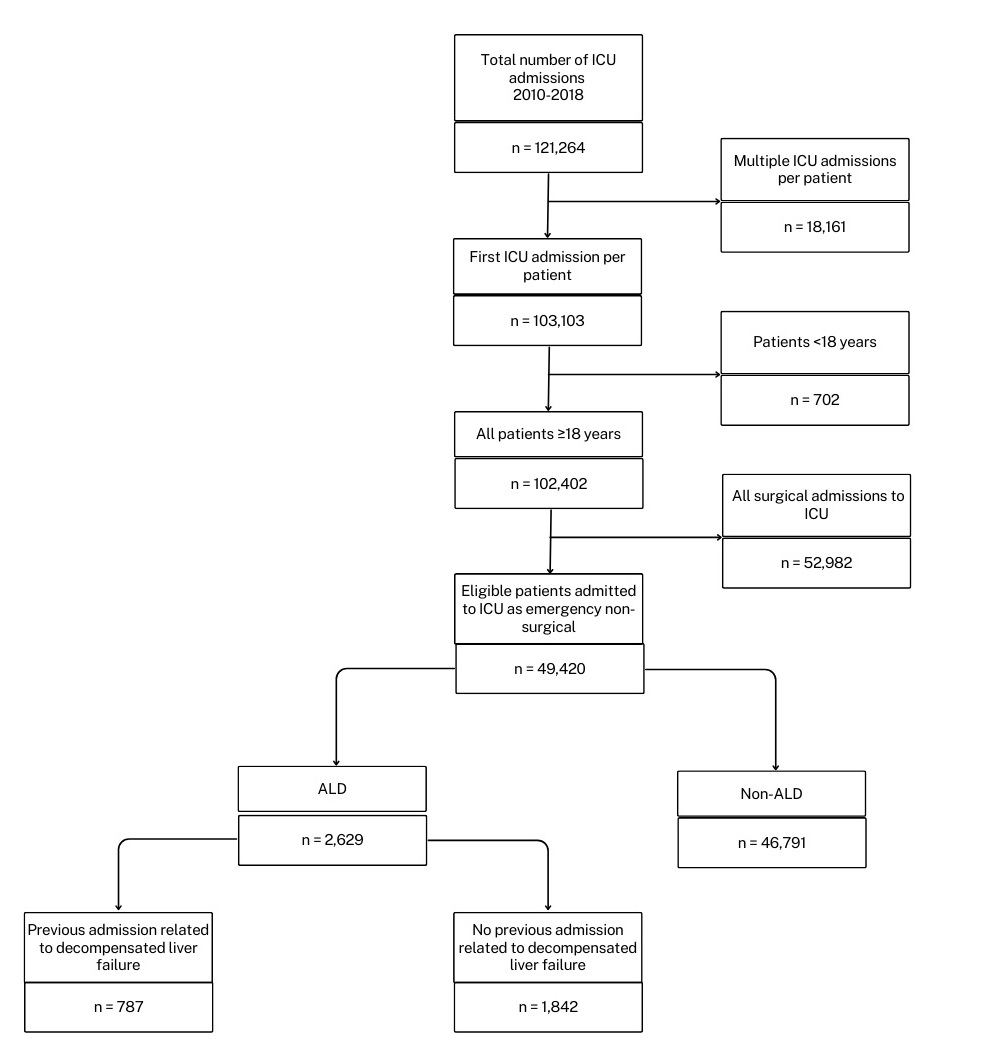


**Figure S2** Plot of all intensive-care unit (ICU) admissions with alcohol-related liver disease (ALD) comorbidity (n=49,420) per 100,000 population plotted over time (01/01/2010-31/12/2018), stratified by Scottish health boards. Smaller health boards were grouped with neighbouring boards, and health boards have been anonymised.


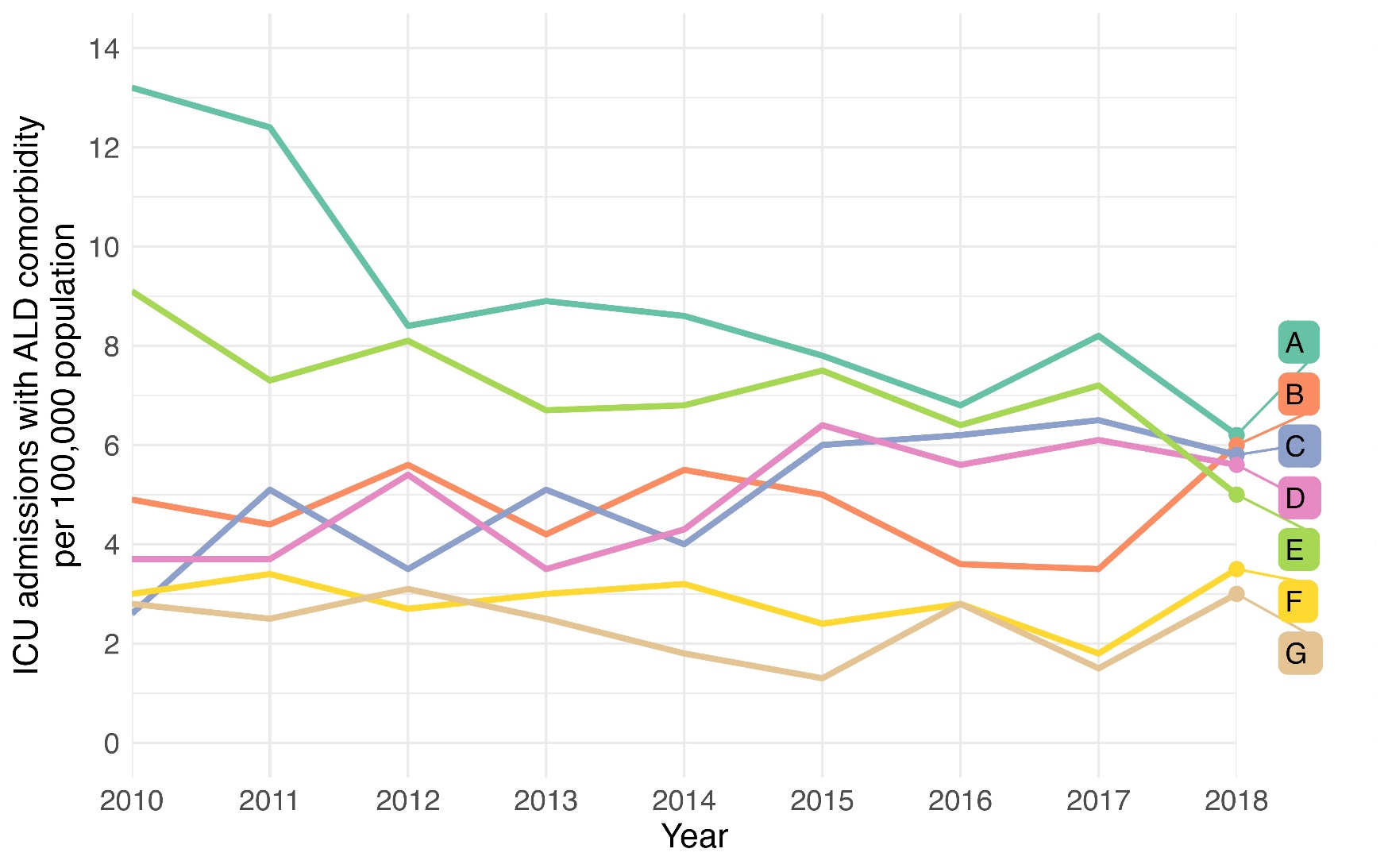


**Figure S3** Kaplan-Meier survival analysis plot of the probability of survival to five years from ICU admission for the ALD cohort (orange, n=2,628) and non-ALD cohort (green, n=46,752) over 01/01/2010-31/12/2018. Probability is summarised as percentage (%).


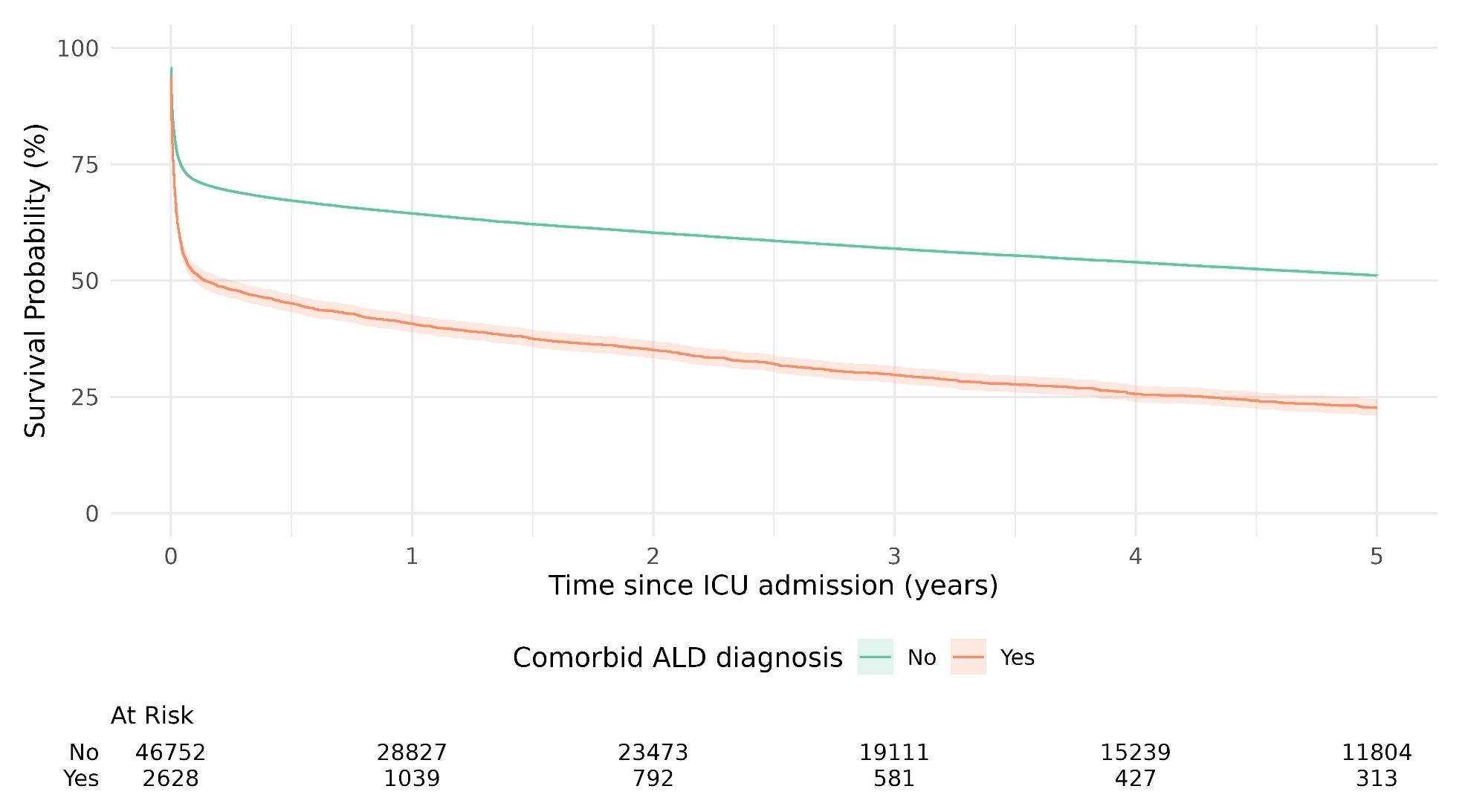


**Figure S4** Cumulative incidence (%) of hospital readmission (hospital survivors only) in ALD (orange, n=1,301) and non-ALD (green, n=32,113) cohorts over two years.

Y-axis cumulative incidence (%) of index hospital readmission per patient, and X-axis denotes time. P value was calculated using Gray’s test.


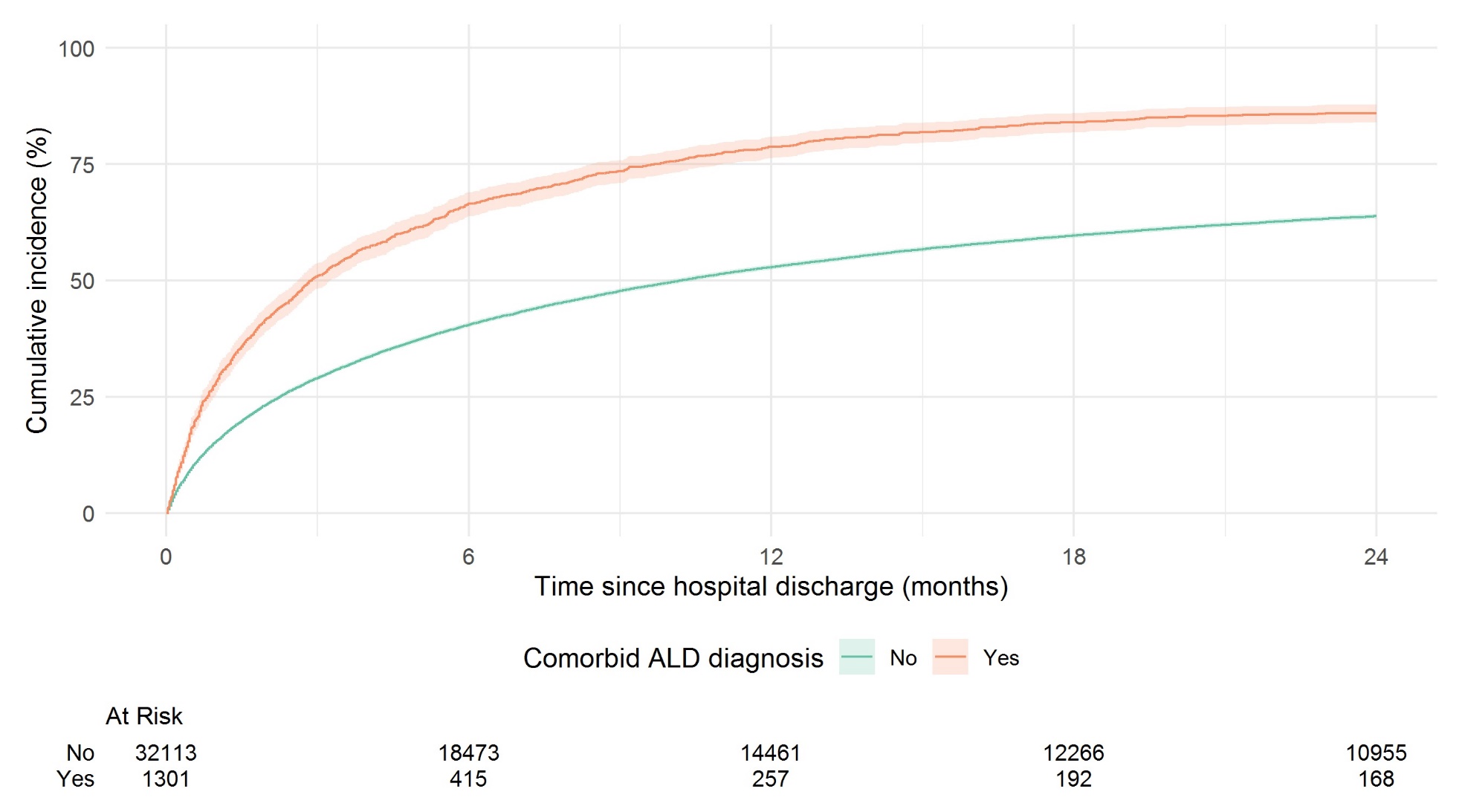


**Figure S5** Subgroup analysis: Kaplan-Meier survival analysis plot of the probability of survival to 60-days from ICU admission for the decompensated ALD cohort (orange, n=1,842) and non-decompensated ALD cohort (green, n=787) over 01/01/2010-31/12/2024. Probability is summarised as percentage (%).


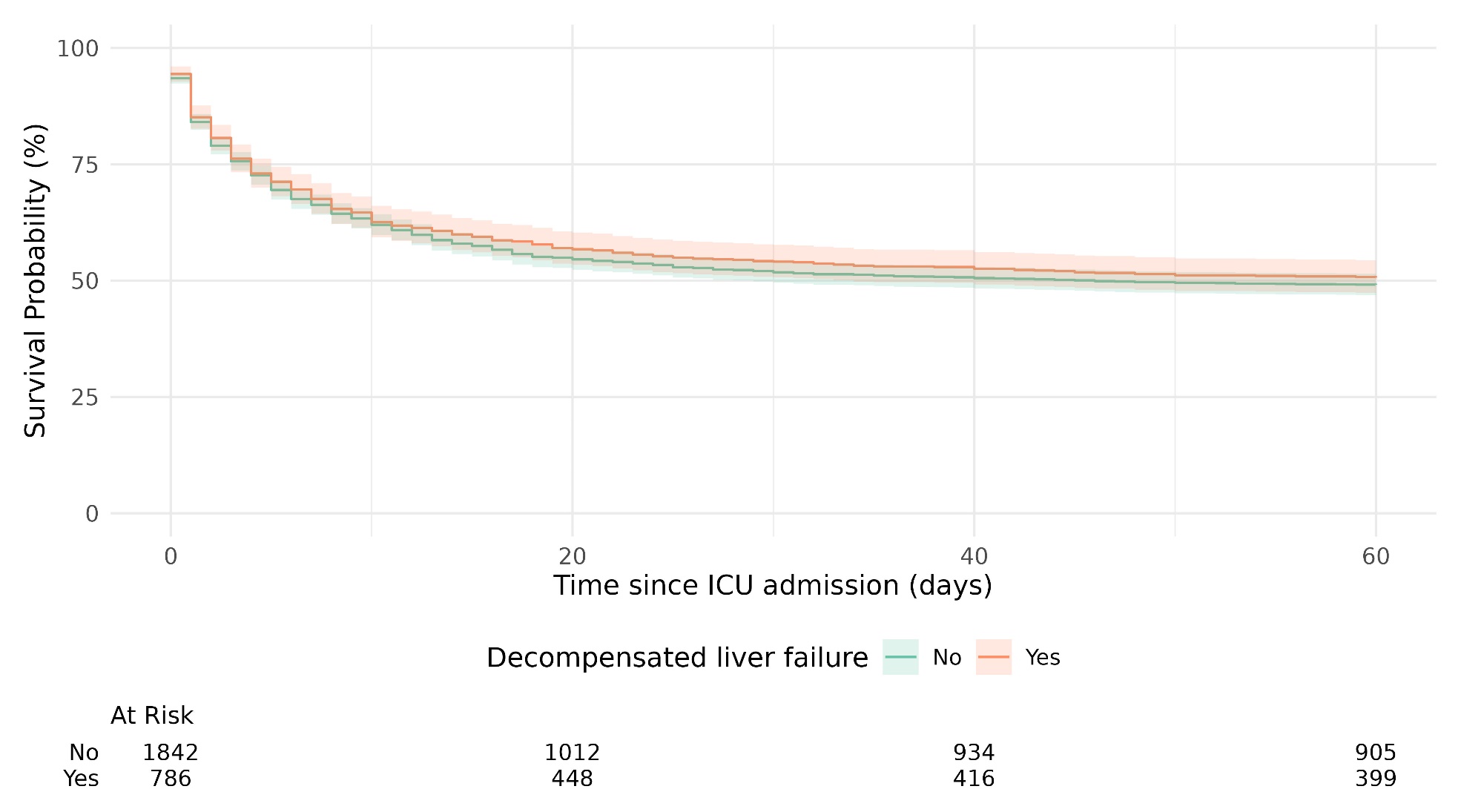


**Figure S6** Subgroup analysis: Kaplan-Meier survival analysis plot of the probability of survival to 60-days from ICU admission for the ALD GI bleed cohort (orange, n=487) and ALD non-GI bleed cohort (green, n=2,133) over 01/01/2010-31/12/2024. Probability is summarised as percentage (%).


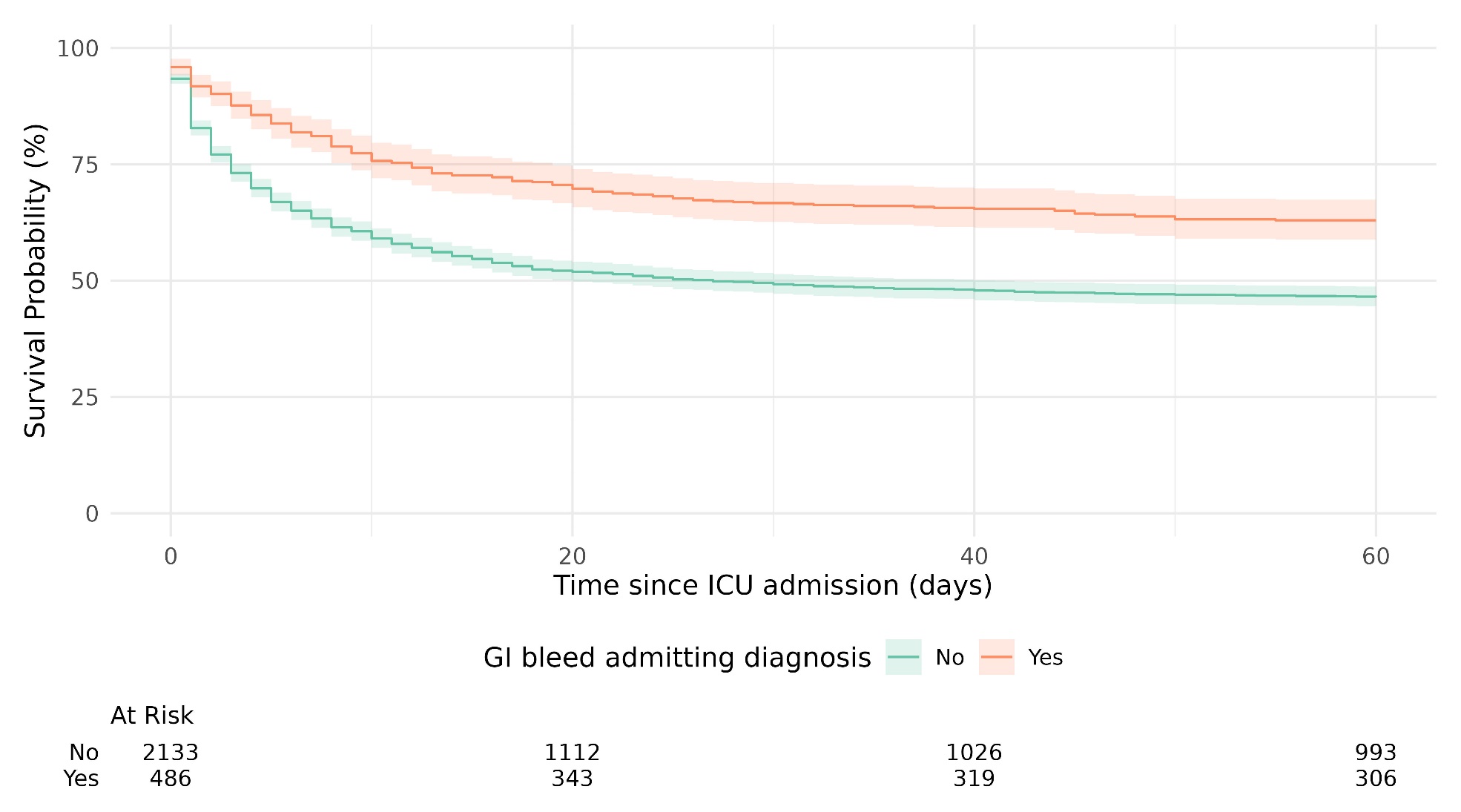


**Figure S7** Sensitivity analysis: Forest plot identifying factors associated with ICU mortality, restricted to only patients with a co-morbid liver disease diagnosis (including ALD) admitted to ICU (n=5,030).

OR: odds ratio; SIMD: Scottish Index of Multiple Deprivation; GI disorder: gastrointestinal disorder; APACHE: Acute Physiology And Chronic Health Evaluation. Number in dataframe = 5030, Number in model = 4965, Missing = 65, AIC = 5488.4, C-statistic = 0.758, H&L = Chi-sq(8) 2­­1.49 (p=0.006)


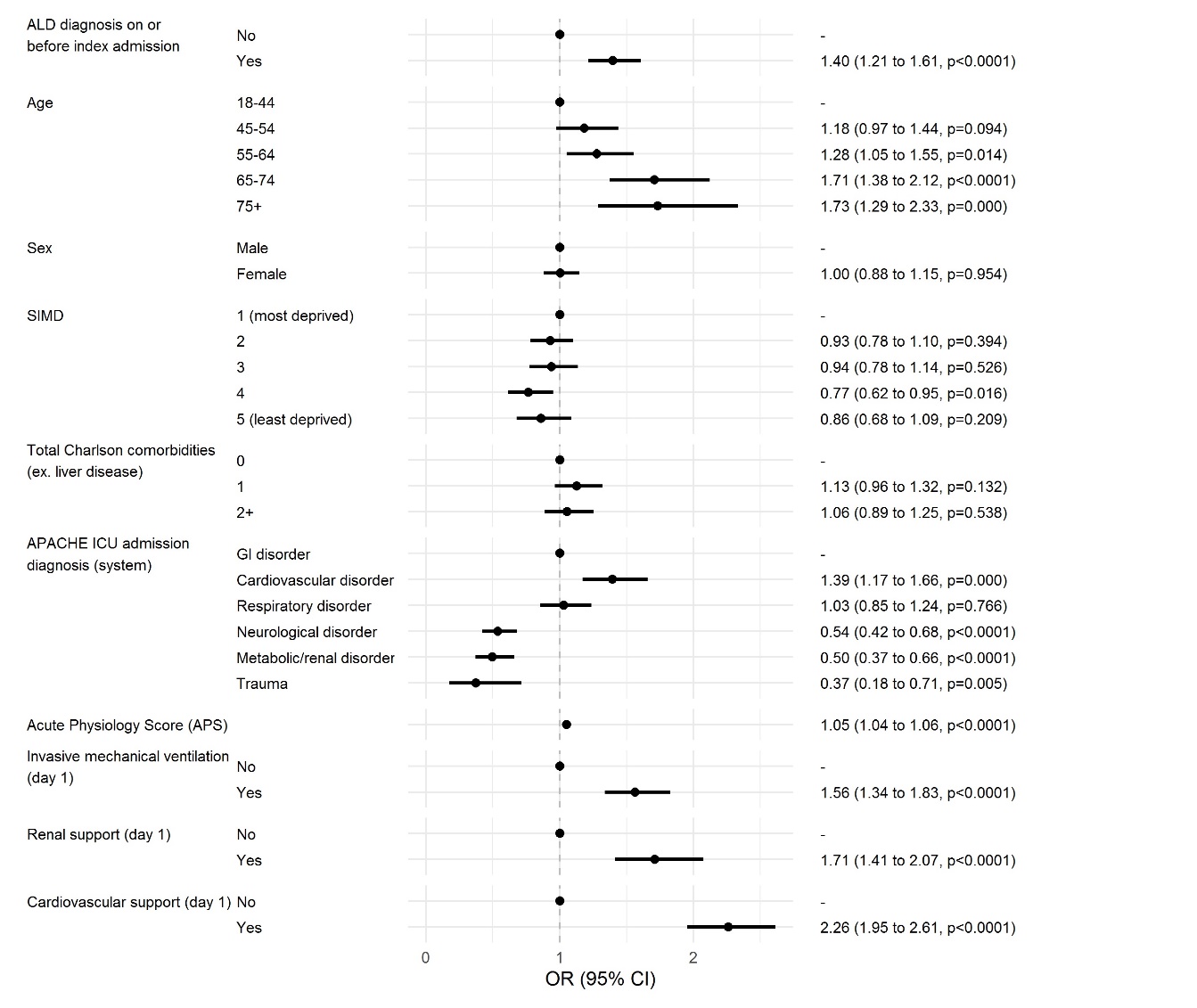


**Figure S8** Sensitivity analysis: Forest plot identifying factors associated with ICU mortality in patients with ALD in ICU, excluding patients with non-ALD liver disease (n=47,019).

OR: odds ratio; SIMD: Scottish Index of Multiple Deprivation; GI disorder: gastrointestinal disorder; APACHE: Acute Physiology And Chronic Health Evaluation. Number in dataframe = 47019, Number in model = 45780, Missing = 1239, AIC = 40252.7, C-statistic = 0.788, H&L = Chi-sq(8) 105.53 (p<0.001). P-values are calculated using the Mann-Whitney U or Kruskal-Wallis test for numerical data and chi-squared test for categorical data. Results are summarized as percentage (%).


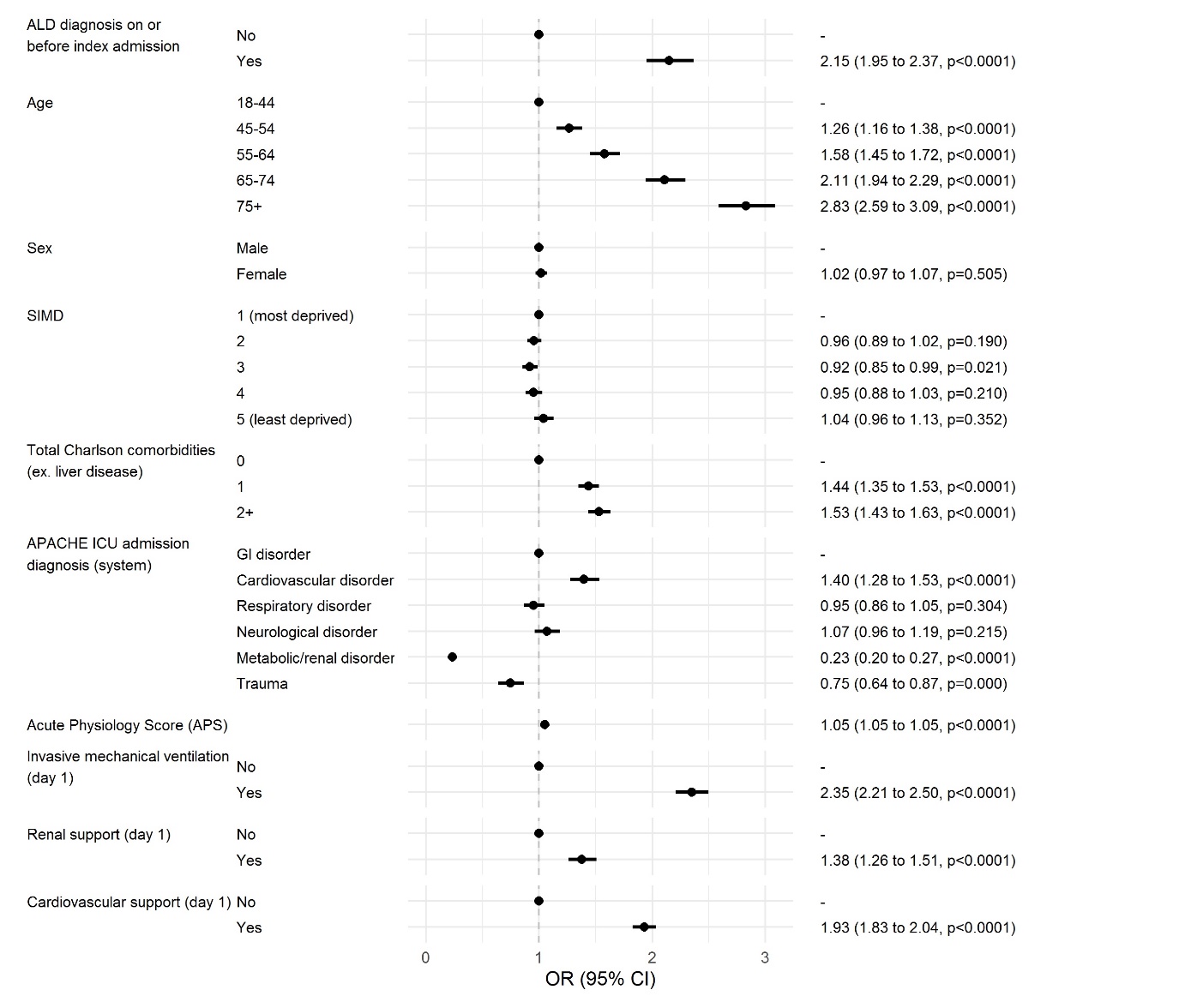

Supplement: Supplementary file 1 — Figure S1. Adult patients with index non‐surgical admission to general ICUs in Scotland stratified by ALD comorbidity. Figure S2. All ICU admissions with alcohol‐related liver disease comorbidity per 100,000 population plotted over time stratified by Scottish health boards. Figure S3. Kaplan–Meier survival analysis plot of the probability of survival to 5 years from ICU admission for the ALD cohort and non‐ALD cohort. Figure S4. Cumulative incidence of hospital readmission (hospital survivors only) in ALD and non‐ALD cohorts. Figure S5. Kaplan–Meier survival analysis plot of the probability of survival to 60‐days from ICU admission for the decompensated ALD cohort and non‐decompensated ALD cohort. Figure S6. Kaplan–Meier survival analysis plot of the probability of survival to 60‐days from ICU admission for the ALD gastrointestinal bleed cohort and ALD non‐ gastrointestinal bleed cohort Figure S7. Forest plot identifying factors associated with ICU mortality, restricted to only patients with a comorbid liver disease diagnosis (including ALD) admitted to ICU. Figure S8. Forest plot identifying factors associated with ICU mortality in patients with ALD in ICU, excluding patients with non‐ALD liver disease. [file ANAE-80-904-s001.docx]
