# Supplementary material for: Outcomes for patients with alcohol‐related liver disease admitted to Scottish intensive care units 2010–2018
Source: Anaesthesia. 2025 Mar 11;80(8):904–14. doi: 10.1111/anae.16599 (PMC12256163; doi:10.1111/anae.16599)
Supplement: Supplementary file 2 — Table S1. Critical care levels. Table S2. ICD‐10 diagnostic codes used to define the ALD comorbidity using the SMR01 database. Table S3. ICD‐10 Diagnostic codes used to define decompensated liver failure group within the ALD cohort. Table S4. ICU admission rate ratios of patients with an ALD comorbidity by Scottish health boards over time Table S5. All ICU admissions with an ALD comorbidity per 100,000 population over time. Table S6. Type of organ support received on day 1 of ICU admission and throughout ICU admission in all patients admitted as emergency non‐surgical to Scottish ICUs. Table S7. Sixty‐day mortality for ALD and non‐ALD groups, stratified by level of organ support. Table S8. Results from the multivariable regression analysis identifying factors associated with ICU mortality in all patients admitted to ICU as an emergency, for non‐surgical reasons. Table S9. Baseline characteristics of patients with ALD with and without a previous hospital admission related to decompensated liver failure 2 years before index ICU admission. Table S10. Summary of treatment in ICU for patients with ALD with and without a previous hospital admission related to decompensated liver failure within 2 years before index ICU admission. Table S11. Mortality rates for patients with ALD with and without a previous hospital admission related to decompensated liver failure in the 2 years before index ICU admission. Table S12.Duration of stay in ICU and hospital for patients with ALD with and without a previous hospital admission related to decompensated liver failure in the 2 years before index ICU admission. Table S13. Readmission within 2 years (hospital survivors only) following index ICU admission for patients with ALD with and without a hospital admission related to decompensated liver failure. Table S14. Baseline characteristics of patients with ALD admitted to ICU with and without a gastrointestinal variceal bleed. Table S15. Summary of treatment and mortality in ICU and hospi [file ANAE-80-904-s002.docx]

**Table S1** Critical Care Levels

| **Levels** | **Critical care** |
| --- | --- |
| Ward care | Patients whose needs can be met through normal ward care, or recently stepped down from higher levels of care requiring support from the critical care outreach team, or at risk of their condition deteriorating. |
| Level 1 | Enhanced care Patients requiring more detailed observation, including basic support for one failing organ system or stepping down from higher levels of care. |
| Level 2 | Critical care  Patients requiring increased levels of observation and support (beyond level 1), including two or more support for failing organ systems or stepping down from higher levels of care. Also includes patients requiring one organ system monitored and supported at an advanced level, or extended post-operative care, or major uncorrected physiological abnormalities or more frequent nursing and therapeutic input. |
| Level 3 | Critical care  Patients requiring advanced respiratory monitoring and support alone, or monitoring and support for two or more failing organ systems at an advanced level, or experience delirium and agitation.  Also includes patients who may have chronic impairment of one or more organ systems, or complex patients requiring multi-organ support. |

(Intensive Care Society). Levels of Care. <https://ics.ac.uk/resource/levels-of-care.html> (accessed 06/02/2024).

**Table S2** ICD-10 diagnostic codes used to define the ALD comorbidity using the SMR01 database

| **ICD-10 Code** | **Definition** |
| --- | --- |
| K70.0 | Alcoholic fatty liver |
| K70.1 | Alcoholic hepatitis |
| K70.2 | Alcoholic fibrosis and sclerosis of liver |
| K70.3 | Alcoholic cirrhosis of liver |
| K70.4 | Alcoholic hepatic failure:   - NOS - Acute - Chronic - Subacute - With or without hepatic coma |
| K70.9 | Alcoholic liver disease, unspecified |

**Table S3** ICD-10 Diagnostic codes used to define decompensated liver failure group within the ALD cohort (n=2,629)

| **ICD-10 Code** | **Definition** |
| --- | --- |
| K70.11 | Alcohol hepatitis with ascites |
| R18.0 | Ascites |
| K72.0 | Acute and subacute hepatic failure, including encephalopathy |
| F10.6 | Wernicke Korsakoff syndrome |
| I85.0 | Oesophageal varices with bleeding |
| I98.3 | Oesophageal varies with bleeding in diseases classified elsewhere, including liver disease |

**Table S4:** ICU admission rate ratios of patients with an ALD comorbidity by Scottish health boards over 01/01/2010-31/12/2018.

| **Health board** | **Admission rate ratio (Year)** |
| --- | --- |
| A | 0.92 (0.89-0.95, p<0.001) |
| B | 0.99 (0.95-1.04, p=0.828) |
| C | 1.08 (1.03-1.13, p=0.001) |
| D* | 1.06 (1.02-1.11, p=0.007) |
| E | 0.96 (0.93-0.98, p=0.001) |
| F | 0.98 (0.94-1.03, p=0.443) |
| G | 0.97 (0.90-1.06, p=0.518) |
| Scotland | 0.98 (0.96-0.99, p=0.005) |
| ICU, intensive care unit; ALD, alcohol-related liver disease.  Admission rate ratios derived from Poisson regression with year entered in the model as a continuous covariate, offset by the population size over time.  * Healthboard experiencing significant reorganisation of funded beds over the study period which may have affected admission rates. | |

**Table S5** All ICU admissions with an ALD comorbidity (n=49,420) per 100,000 population over time (01/01/2010-31/12/2018).

| **Health Board** | **2010** | **2011** | **2012** | **2013** | **2014** | **2015** | **2016** | **2017** | **2018** |
| --- | --- | --- | --- | --- | --- | --- | --- | --- | --- |
| A | 13.2 | 12.4 | 8.4 | 8.9 | 8.6 | 7.8 | 6.8 | 8.2 | 6.2 |
| B | 4.9 | 4.4 | 5.6 | 4.2 | 5.5 | 5.0 | 3.6 | 3.5 | 6.0 |
| C | 2.6 | 5.1 | 3.5 | 5.1 | 4.0 | 6.0 | 6.2 | 6.5 | 5.8 |
| D* | 3.7 | 3.7 | 5.4 | 3.5 | 4.3 | 6.4 | 5.6 | 6.1 | 5.6 |
| E | 9.1 | 7.3 | 8.1 | 6.7 | 6.8 | 7.5 | 6.4 | 7.2 | 5.0 |
| F | 3.0 | 3.4 | 2.7 | 3.0 | 3.2 | 2.4 | 2.8 | 1.8 | 3.5 |
| G | 2.8 | 2.5 | 3.1 | 2.5 | 1.8 | 1.3 | 2.8 | 1.5 | 3.0 |
| Scotland | 6.2 | 6.0 | 5.6 | 5.2 | 5.2 | 5.5 | 5.1 | 5.3 | 5.1 |
| ICU, intensive-care unit; ALD, alcohol-related liver disease.  * Healthboard experiencing significant reorganisation of funded beds over the study period which may have affected admission rates. | | | | | | | | | |

**Table S6:** Type of organ support received on day one of ICU admission and throughout ICU admission in all patients admitted as emergency non-surgical to Scottish ICUs (n=49,420) over 01/01/2010-31/12/2018. P-values were calculated using the Mann-Whitney test, Kruskal-Wallis or χ2-test. Results are summarized as percentage (%).

| **Outcome** |  | **Non-ALD (n=46,791)** | **ALD (n=2,629)** | **Total (n=49,420)** | **p-value** |
| --- | --- | --- | --- | --- | --- |
| IMV support (day 1) | No | 18072 (38.6) | 737 (28.1) | 18809 (38.1) | <0.0001 |
|  | Yes | 28689 (61.4) | 1889 (71.9) | 30578 (61.9) |  |
|  | (Missing) | * | * | 33 |  |
| Renal support (day 1) | No | 43315 (92.6) | 2348 (89.4) | 45663 (92.5) | <0.0001 |
|  | Yes | 3446 (7.4) | 278 (10.6) | 3724 (7.5) |  |
|  | (Missing) | * | * | 33 |  |
| Cardiovascular support (day 1) | No | 27152 (58.1) | 1411 (53.7) | 28563 (57.8) | <0.0001 |
|  | Yes | 19609 (41.9) | 1215  (46.3) | 20824 (42.2) |  |
|  | (Missing) | * | * | 33 |  |
| IMV support (any time across admission) | No | 15521 (33.2) | 569 (21.7) | 16090 (32.6) | <0.0001 |
|  | Yes | 31252 (66.8) | 2058 (78.3) | 33310 (67.4) |  |
|  | (Missing) | * | * | 20 |  |
| Renal support (any time across admission) | No | 40512 (86.6) | 2147 (81.7) | 42659 (86.4) | <0.0001 |
|  | Yes | 6261 (13.4) | 480 (18.3) | 6741 (13.6) |  |
|  | (Missing) | * | * | 20 |  |
| Cardiovascular support (any time across admission) | No | 22246 (47.6) | 1069 (40.7) | 23315 (47.2) | <0.0001 |
|  | Yes | 24527  (52.4) | 1558  (59.3) | 26085 (52.8) |  |
|  | (Missing) | * | * | 20 |  |
| Total days with any organ support | Median (IQR) | 2.0  (1.0 to 4.0) | 2.0  (1.0 to 6.0) | 2.0  (1.0 to 4.0) | <0.0001 |
| Maximum number of organs supported across admission | 0 | 10570 (22.6) | 371 (14.1) | 10941 (22.1) | <0.0001 |
|  | 1 | 15031 (32.1) | 802 (30.5) | 15833 (32.1) |  |
|  | 2 | 16507 (35.3) | 1068 (40.7) | 17575 (35.6) |  |
|  | 3 | 4665 (10.0) | 386 (14.7) | 5051 (10.2) |  |
|  | (Missing) | * | * | 20 |  |
| ALD, alcohol-related liver disease; ICU, intensive care unit; IMV, invasive mechanical ventilation.  * Values <10 suppressed due to disclosure risk.  Range is not reported with median (IQR) due to disclosure risk for values <10. | | | | | |

**Table S7:** 60-day mortality for ALD and non-ALD groups, stratified by level of organ support.

Results are summarized as percentage (%).

|  |  | **Organs supported (day 1)** | | | |  |
| --- | --- | --- | --- | --- | --- | --- |
| **ALD** | **60-day mortality** | **0** | **1** | **2** | **3** | **Total** |
| Non-ALD (n=46,722, 69 missing) | Survivors | 11146 (85.3) | 13200 (75.0) | 7721 (54.9) | 787 (39.7) | 32854 (70.3) |
|  | Non-Survivors | 1922 (14.7) | 4399 (25.0) | 6352 (45.1) | 1195 (60.3) | 13868 (29.7) |
| ALD (n=2,625, 4 missing) | Survivors | 330 (64.0) | 627 (60.6) | 309 (35.2) | 34 (17.3) | 1300 (49.5) |
|  | Non-Survivors | 186 (36.0) | 407 (39.4) | 570 (64.8) | 162 (82.7) | 1325 (50.5) |
| ALD, alcohol-related liver disease; IMV, invasive mechanical ventilation. | | | | | | |

**Table S8:** Results from the multi-variable regression analysis identifying factors associated with ICU mortality in all emergency, non-surgical patients admitted to ICU (n=49,420). P-values are calculated using the Mann-Whitney U test or Kruskal-Wallis test for numerical data and χ2-squared for categorical data. Results are summarized as percentage (%) or odds ratio (CI, p-value).

| **Dependent: ICU mortality** |  | **Survivors** | **Non-Survivors** | **OR (univariable)** | **OR (multivariable)** |
| --- | --- | --- | --- | --- | --- |
| ALD diagnosis on or before index admission | No | 36222 (77.5) | 10517 (22.5) | - | - |
|  | Yes | 1664 (63.3) | 964 (36.7) | 2.00 (1.84-2.17, p<0.0001) | 2.03 (1.85-2.24, p<0.0001) |
| Age | 18-44 | 10879 (88.1) | 1472 (11.9) | - | - |
|  | 45-54 | 6667 (80.3) | 1637 (19.7) | 1.81 (1.68-1.96, p<0.0001) | 1.27 (1.17-1.39, p<0.0001) |
|  | 55-64 | 7409 (74.5) | 2536 (25.5) | 2.53 (2.36-2.72, p<0.0001) | 1.58 (1.45-1.71, p<0.0001) |
|  | 65-74 | 7502 (69.7) | 3265 (30.3) | 3.22 (3.01-3.44, p<0.0001) | 2.09 (1.93-2.26, p<0.0001) |
|  | 75+ | 5429 (67.9) | 2571 (32.1) | 3.50 (3.26-3.76, p<0.0001) | 2.74 (2.51-2.98, p<0.0001) |
| Sex | Male | 21463 (76.6) | 6572 (23.4) | - | - |
|  | Female | 16222 (76.9) | 4877 (23.1) | 0.98 (0.94-1.02, p=0.3956) | 1.02 (0.97-1.07, p=0.5235) |
| SIMD | 1 (most deprived) | 10951 (76.9) | 3281 (23.1) | - | - |
|  | 2 | 9279 (77.3) | 2723 (22.7) | 0.98 (0.92-1.04, p=0.4823) | 0.94 (0.88-1.01, p=0.0751) |
|  | 3 | 7053 (76.8) | 2134 (23.2) | 1.01 (0.95-1.07, p=0.7567) | 0.92 (0.86-0.99, p=0.0234) |
|  | 4 | 5463 (76.1) | 1720 (23.9) | 1.05 (0.98-1.12, p=0.1453) | 0.93 (0.87-1.01, p=0.0713) |
|  | 5 (least deprived) | 4333 (74.6) | 1472 (25.4) | 1.13 (1.06-1.22, p=0.0005) | 1.01 (0.93-1.10, p=0.7826) |
| Total Charlson comorbidities (ex. liver disease) | 0 | 15723 (84.0) | 2990 (16.0) | - | - |
|  | 1 | 11905 (74.4) | 4099 (25.6) | 1.81 (1.72-1.91, p<0.0001) | 1.43 (1.35-1.52, p<0.0001) |
|  | 2+ | 10057 (69.8) | 4360 (30.2) | 2.28 (2.16-2.40, p<0.0001) | 1.52 (1.43-1.62, p<0.0001) |
| APACHE ICU admission diagnosis (system) | GI disorder | 3605 (75.0) | 1200 (25.0) | - | - |
|  | Cardiovascular disorder | 9540 (64.5) | 5244 (35.5) | 1.65 (1.53-1.78, p<0.0001) | 1.31 (1.21-1.43, p<0.0001) |
|  | Respiratory disorder | 9757 (78.4) | 2682 (21.6) | 0.83 (0.76-0.89, p<0.0001) | 0.89 (0.82-0.97, p=0.0112) |
|  | Neurological disorder | 5951 (78.2) | 1662 (21.8) | 0.84 (0.77-0.91, p=0.0001) | 0.97 (0.88-1.07, p=0.5332) |
|  | Metabolic/renal disorder | 5987 (94.7) | 335 (5.3) | 0.17 (0.15-0.19, p<0.0001) | 0.23 (0.20-0.26, p<0.0001) |
|  | Trauma | 2860 (90.0) | 319 (10.0) | 0.34 (0.29-0.38, p<0.0001) | 0.68 (0.59-0.79, p<0.0001) |
| Acute Physiology Score (APS) | Mean (SD) | 12.9 (7.6) | 18.9 (10.8) | 1.08 (1.08-1.09, p<0.0001) | 1.05 (1.05-1.05, p<0.0001) |
| Invasive mechanical ventilation (day 1) | No | 16627 (88.4) | 2173 (11.6) | - | - |
|  | Yes | 21239 (69.6) | 9295 (30.4) | 3.35 (3.18-3.52, p<0.0001) | 2.32 (2.19-2.46, p<0.0001) |
| Renal support (day 1) | No | 35697 (78.3) | 9915 (21.7) | - | - |
|  | Yes | 2169 (58.3) | 1553 (41.7) | 2.58 (2.41-2.76, p<0.0001) | 1.45 (1.34-1.58, p<0.0001) |
| Cardiovascular support (day 1) | No | 24841 (87.0) | 3699 (13.0) | - | - |
|  | Yes | 13025 (62.6) | 7769 (37.4) | 4.01 (3.83-4.19, p<0.0001) | 1.93 (1.83-2.03, p<0.0001) |
| ALD: alcohol-related liver disease; SIMD: Scottish Index of Multiple Deprivation; APACHE: Acute Physiology And Chronic Health Evaluation; OR: odds ratio; APS: Acute Physiology Score; GI disorder: gastrointestinal disorder; SD: standard deviation. | | | | | |

**Table S9**: Subgroup analysis: Baseline characteristics of patients with ALD with and without a previous hospital admission related to decompensated liver failure 2 years prior to their index ICU admission (n=2,629). P-value is calculated using the Mann-Whitney U test or Kruskal-Wallis test for numerical data and χ2-squared test for categorical data. Results are summarized as percentage (%) or median (IQR).

| **Outcome** |  | **No decompensated liver failure (n=1,842)** | **Decompensated liver failure (n=787)** | **Total (n=2629)** | **p-value** |
| --- | --- | --- | --- | --- | --- |
| Age (continuous) | Median (IQR) | 54.0 (46.0 to 62.0) | 52.0 (43.0 to 58.0) | 54.0 (46.0 to 61.0) | <0.0001 |
| Sex | Male | 1147 (62.3) | 519 (65.9) | 1666 (63.4) | 0.0804 |
|  | Female | 695 (37.7) | 268 (34.1) | 963 (36.6) |  |
| SIMD | 1 (most deprived) | 706 (38.7) | 291 (37.2) | 997 (38.2) | 0.2020 |
|  | 2 | 441 (24.2) | 215 (27.5) | 656 (25.2) |  |
|  | 3 | 304 (16.7) | 115 (14.7) | 419 (16.1) |  |
|  | 4 | 224 (12.3) | 86 (11.0) | 310 (11.9) |  |
|  | 5 (least deprived) | 150 (8.2) | 75 (9.6) | 225 (8.6) |  |
|  | (Missing) | * | * | 22 |  |
| Total Charlson comorbidities (ex. liver disease) | 0 | 884 (48.0) | 321 (40.8) | 1205 (45.8) | 0.0026 |
|  | 1 | 585 (31.8) | 277 (35.2) | 862 (32.8) |  |
|  | 2+ | 373 (20.2) | 189 (24.0) | 562 (21.4) |  |
| APACHE ICU admission diagnosis (system) | GI disorder | 587 (32.0) | 323 (41.1) | 910 (34.7) | <0.0001 |
|  | Cardiovascular disorder | 383 (20.9) | 146 (18.6) | 529 (20.2) |  |
|  | Respiratory disorder | 416 (22.7) | 119 (15.2) | 535 (20.4) |  |
|  | Neurological disorder | 258 (14.1) | 128 (16.3) | 386 (14.7) |  |
|  | Metabolic/renal disorder | 145 (7.9) | 53 (6.8) | 198 (7.6) |  |
|  | Trauma | 46 (2.5) | 16 (2.0) | 62 (2.4) |  |
|  | (Missing) | * | * | * |  |
| APACHE ICU admission diagnosis (5 most frequent for ALD patients) | Respiratory infection | 249 (13.5) | 65 (8.3) | 314 (11.9) | <0.0001 |
|  | Sepsis | 215 (11.7) | 84 (10.7) | 299 (11.4) |  |
|  | Other gastrointestinal disorder† | 310 (16.8) | 111 (14.1) | 421 (16.0) |  |
|  | ICH/SDH/SAH | 123 (6.7) | 47 (6.0) | 170 (6.5) |  |
|  | GI bleeding | 276 (15.0) | 211 (26.8) | 487 (18.5) |  |
|  | Other | 669 (36.3) | 269 (34.2) | 938 (35.7) |  |
| Acute Physiology Score (APS) | Median (IQR) | 18.0 (11.0 to 24.0) | 17.0 (11.0 to 23.0) | 18.0 (11.0 to 24.0) | 0.0907 |
| APACHE II Score | Median (IQR) | 23.0 (16.0 to 29.0) | 23.0 (17.0 to 29.0) | 23.0 (16.0 to 29.0) | 0.7049 |
| Bilirubin; umol.l^-1^ (continuous) | Median (IQR) | 50.0 (18.0 to 112.8) | 64.0 (31.0 to 114.5) | 55.0 (21.0 to 113.0) | 0.0001 |
| SIMD: Scottish Index of Multiple Deprivation; ICU: Intensive-care unit; GI: Gastro-intestinal; IQR: interquartile range; APACHE: Acute Physiology And Chronic Health Evaluation.  † Other GI disorder constitutes of pancreatitis, GI perforation/rupture, cholangitis/cholecystitis, GI obstruction, hepatic failure-toxin, GI vascular insufficiency/embolism/infarction, hepatic failure-overdose, GI neoplasm, localised GI abscess/cyst, peritonitis, GI inflammatory disease, hepatic failure-virus, hepatic failure-drug reaction, diverticulosis, acute corrosive injury.  * Values <10 suppressed due to disclosure risk.  Range is not reported with median (IQR) due to disclosure risk for values <10. | | | | | |

**Table S10** Subgroup analysis: Summary of treatment in ICU for patients with ALD with and without a previous hospital admission related to decompensated liver failure within 2 years prior to their index ICU admission (n=2,629). P-values are calculated using the Mann-Whitney U test or Kruskal-Wallis test. Results are summarized as percentage (%).

| **Outcome** |  | **No decompensated liver failure (n=1,842)** | **Decompensated liver failure (n=787)** | **Total**  **(n=2,629)** | **p-value** |
| --- | --- | --- | --- | --- | --- |
| IMV support (day 1) | No | 524  (28.5) | 213  (27.1) | 737  (28.1) | 0.5010 |
|  | Yes | 1316  (71.5) | 573  (72.9) | 1889  (71.9) |  |
|  | (Missing) | * | * | * |  |
| Renal support (day 1) | No | 1642  (89.2) | 706  (89.8) | 2348  (89.4) | 0.7075 |
|  | Yes | 198  (10.8) | 80  (10.2) | 278  (10.6) |  |
|  | (Missing) | * | * | * |  |
| Cardiovascular support (day 1) | No | 952  (51.7) | 459  (58.4) | 1411  (53.7) | 0.0020 |
|  | Yes | 888  (48.3) | 327  (41.6) | 1215  (46.3) |  |
|  | (Missing) | * | * | * |  |
| IMV support (any time across admission) | No | 401  (21.8) | 168  (21.3) | 569  (21.7) | 0.8393 |
|  | Yes | 1439  (78.2) | 619  (78.7) | 2058  (78.3) |  |
|  | (Missing) | * | * | * |  |
| Renal support (any time across admission) | No | 1485  (80.7) | 662  (84.1) | 2147  (81.7) | 0.0437 |
|  | Yes | 355  (19.3) | 125  (15.9) | 480  (18.3) |  |
|  | (Missing) | * | * | * |  |
| Cardiovascular support (any time across admission) | No | 705  (38.3) | 364  (46.3) | 1069  (40.7) | 0.0002 |
|  | Yes | 1135  (61.7) | 423  (53.7) | 1558  (59.3) |  |
|  | (Missing) | * | * | * |  |
| Total days with any organ support | Median (IQR) | 2.0  (1.0 to 6.0) | 2.0  (1.0 to 4.0) | 2.0  (1.0 to 6.0) | 0.0049 |
| Maximum number of organs supported across admission | 0 | 255  (13.9) | 116  (14.7) | 371  (14.1) | 0.0065 |
|  | 1 | 529  (28.8) | 273  (34.7) | 802  (30.5) |  |
|  | 2 | 768  (41.7) | 300  (38.1) | 1068  (40.7) |  |
|  | 3 | 288  (15.7) | 98  (12.5) | 386  (14.7) |  |
|  | (Missing) | * | * | * |  |
| IQR: interquartile range; IMV: Invasive mechanical ventilation.  * Values <10 suppressed due to disclosure risk.  Range is not reported with median (IQR) due to disclosure risk for values <10. | | | | | |

**Table S11** Subgroup analysis: Mortality rates for patients with ALD with and without a previous hospital admission related to decompensated liver failure in the 2 years prior to their index ICU admission (n=2,629). P-value is calculated using the χ2-squared test. Results are summarized as percentage (%).

| **Outcome** |  | **No decompensated liver failure (n=1,842)** | **Decompensated liver failure (n=787)** | **Total (n=2,629)** | **p-value** |
| --- | --- | --- | --- | --- | --- |
| ICU mortality | Survivors | 1135  (61.6) | 529  (67.3) | 1664 (63.3) | 0.0064 |
|  | Non-Survivors | 707  (38.4) | 257  (32.7) | 964  (36.7) |  |
|  | (Missing) | * | * | * |  |
| Hospital mortality | Survivors | 899  (49.3) | 402  (51.9) | 1301 (50.1) | 0.2329 |
|  | Non-Survivors | 925  (50.7) | 372 (48.1) | 1297 (49.9) |  |
|  | (Missing) | 18 | 13 | 31 |  |
| 60-day mortality | Survivors | 904  (49.1) | 398  (50.6) | 1302 (49.5) | 0.4906 |
|  | Non-Survivors | 938  (50.9) | 388  (49.4) | 1326 (50.5) |  |
|  | (Missing) | * | * | * |  |
| Two-year mortality | Survivors | 577 (33.4) | 215 (28.4) | 792 (31.9) | 0.0173 |
|  | Non-Survivors | 1152 (66.6) | 541 (71.6) | 1693 (68.1) |  |
|  | (Missing) | 113 | 31 | 144 |  |
| Five-year mortality | Survivors | 238 (15.4) | 75 (10.8) | 313 (86.0) | 0.0046 |
|  | Non-Survivors | 1307 (84.6) | 619 (89.2) | 1926 (86.0) |  |
|  | (Missing) | 297 | 93 | 390 |  |
| ICU, intensive-care unit; ALD, alcohol-related liver disease.  * Values <10 suppressed due to disclosure risk. | | | | | |

**Table S12** Subgroup analysis: Length of stay in ICU and hospital for patients with ALD with and without a previous hospital admission related to decompensated liver failure in the 2 years prior to their index ICU admission (n=2,629). Length of stay is stratified according to ICU and hospital survivorship. P-value is calculated using the Mann-Whitney U test. Results are summarized as median (IQR).

| **Outcome** |  | **No decompensated liver failure (n=1,135)** | **Decompensated liver failure (n=529)** | **Total (n=1,664)** | **p-value** |
| --- | --- | --- | --- | --- | --- |
| ICU length of stay (ICU survivors) | Median (IQR) | 3.0 (1.0 to 8.0) | 3.0 (1.0 to 6.0) | 3.0 (1.0 to 7.0) | 0.0097 |

| **Outcome** |  | **No decompensated liver failure (n=707)** | **Decompensated liver failure (n=257)** | **Total (n=964)** | **p-value** |
| --- | --- | --- | --- | --- | --- |
| ICU length of stay (ICU non-survivors) | Median (IQR) | 2.0 (1.0 to 6.0) | 2.0 (1.0 to 4.0) | 2.0 (1.0 to 5.0) | 0.0667 |

| **Outcome** |  | **No decompensated liver failure (n=899)** | **Decompensated liver failure (n=402)** | **Total (n=1,301)** | **p-value** |
| --- | --- | --- | --- | --- | --- |
| Hospital length of stay (Hospital survivors) | Median (IQR) | 17.0 (8.0 to 32.0) | 14.0 (7.0 to 29.0) | 16.0 (8.0 to 31.0) | 0.0016 |

| **Outcome** |  | **No decompensated liver failure (n=925)** | **Decompensated liver failure (n=372)** | **Total (n=1,297)** | **p-value** |
| --- | --- | --- | --- | --- | --- |
| Hospital length of stay (Hospital non-survivors) | Median (IQR) | 4.0 (1.0 to 10.0) | 3.5 (1.0 to 10.0) | 4.0 (1.0 to 10.0) | 0.8991 |
| ICU, intensive care unit; IQR, interquartile range.  Range is not reported with median (IQR) due to disclosure risk for values <10. | | | | | |

**Table S13** Subgroup analysis: Readmission within two years (hospital survivors only) following index ICU admission for patients with ALD with and without a hospital admission related to decompensated liver failure (n=1,301). P value was calculated using the χ2-squared test. Results are summarized as number (%).

| **Outcome** |  | **No decompensated liver failure (n=899)** | **Decompensated liver failure (n=402)** | **Total**  **(n=1,301)** | **p-value** |
| --- | --- | --- | --- | --- | --- |
| ICU readmission within 2 years | No | 646 (81.7) | 268 (71.3) | 914 (78.3) | 0.0001 |
|  | Yes | 145 (18.3) | 108 (28.7) | 253 (21.7) |  |
|  | (Missing) | 108 | 26 | 134 |  |
| Hospital readmission within 2 years | No | 142 (16.4) | 37 (9.2) | 179 (14.2) | 0.0009 |
|  | Yes | 723 (83.6) | 363 (90.8) | 1086 (85.8) |  |
|  | (Missing) | * | * | 36 |  |
| ICU, intensive-care unit.  * Values <10 suppressed due to disclosure risk. | | | | | |

**Table S14:** Subgroup analysis: Baseline characteristics of patients with ALD admitted to ICU with and without a GI variceal bleed (n=2,620). P-value is calculated using the Mann-Whitney U test or Kruskal-Wallis test for numerical data and χ2-squared test for categorical data. Results are summarized as percentage (%) or median (IQR).

| **Variable** |  | **GI bleeding: No (n=2,133)** | **GI bleeding: Yes (n=487)** | **Total (n=2620)** | **p-value** |
| --- | --- | --- | --- | --- | --- |
| Age (continuous) | Median (IQR) | 54.0 (46.0 to 61.0) | 53.0 (45.0 to 61.0) | 54.0 (46.0 to 61.0) | 0.9430 |
| Sex | Male | 1323 (62.0) | 338 (69.4) | 1661 (63.4) | 0.0027 |
|  | Female | 810 (38.0) | 149 (30.6) | 959 (36.6) |  |
|  | (Missing) | * | * | * |  |
| SIMD | 1 (most deprived) | 857 (40.5) | 135 (28.0) | 992 (38.2) | <0.0001 |
|  | 2 | 523 (24.7) | 132 (27.4) | 655 (25.2) |  |
|  | 3 | 336 (15.9) | 81 (16.8) | 417 (16.0) |  |
|  | 4 | 243 (11.5) | 67 (13.9) | 310 (11.9) |  |
|  | 5 (least deprived) | 158 (7.5) | 67 (13.9) | 225 (8.7) |  |
|  | (Missing) | * | * | 21 |  |
| Total Charlson comorbidities (ex. liver disease) | 0 | 954 (44.7) | 247 (50.7) | 1201 (45.8) | 0.0310 |
|  | 1 | 705 (33.1) | 153 (31.4) | 858 (32.7) |  |
|  | 2+ | 474 (22.2) | 87 (17.9) | 561 (21.4) |  |
|  | (Missing) | * | * | * |  |
| Acute Physiology Score (APS) | Median (IQR) | 19.0 (13.0 to 24.0) | 12.0 (8.0 to 18.0) | 18.0 (11.0 to 24.0) | <0.0001 |
| APACHE II Score | Median (IQR) | 24.0 (17.0 to 30.0) | 19.0 (14.0 to 25.0) | 23.0 (16.0 to 29.0) | <0.0001 |
| Bilirubin; umol.l^-1^ (continuous) | Median (IQR) | 50.0 (19.0 to 112.0) | 70.0 (38.0 to 117.5) | 55.5 (21.0 to 114.0) | <0.0001 |
| SIMD, Scottish Index of Multiple Deprivation; ICU, Intensive-care unit; IQR, interquartile range; APACHE, Acute Physiology And Chronic Health Evaluation.  * Values <10 suppressed due to disclosure risk.  Range is not reported with median (IQR) due to disclosure risk for values <10. | | | | | |

**Table S15:** Summary of treatment and mortality in ICU and hospital for patients with ALD admitted to ICU with and without a GI variceal bleed (n=2,620). P-value is calculated using the Mann-Whitney U test or Kruskal-Wallis test for numerical data and χ2-squared test for categorical data. Results are summarized as percentage (%).

| **Outcome** |  | **GI bleeding: No (n=2,133)** | **GI bleeding: Yes (n=487)** | **Total (n=2620)** | **p-value** |
| --- | --- | --- | --- | --- | --- |
| Invasive mechanical ventilation (day 1) | No | 635 (29.8) | 100 (20.5) | 735 (28.1) | 0.0001 |
|  | Yes | 1495 (70.2) | 387 (79.5) | 1882 (71.9) |  |
|  | (Missing) | * | * | * |  |
| Renal support (day 1) | No | 1871 (87.8) | 468 (96.1) | 2339 (89.4) | <0.0001 |
|  | Yes | 259 (12.2) | 19 (3.9) | 278 (10.6) |  |
|  | (Missing) | * | * | * |  |
| Cardiovascular support (day 1) | No | 1048 (49.2) | 359 (73.7) | 1407 (53.8) | <0.0001 |
|  | Yes | 1082 (50.8) | 128 (26.3) | 1210 (46.2) |  |
|  | (Missing) | * | * | * |  |
| IMV (any time across admission) | No | 484 (22.7) | 83 (17.0) | 567 (21.7) | 0.0074 |
|  | Yes | 1647 (77.3) | 404 (83.0) | 2051 (78.3) |  |
|  | (Missing) | * | * | * |  |
| Renal support (any time across admission) | No | 1683 (79.0) | 455 (93.4) | 2138 (81.7) | <0.0001 |
|  | Yes | 448 (21.0) | 32 (6.6) | 480 (18.3) |  |
|  | (Missing) | * | * | * |  |
| Cardiovascular support (any time across admission) | No | 759 (35.6) | 307 (63.0) | 1066 (40.7) | <0.0001 |
|  | Yes | 1372 (64.4) | 180 (37.0) | 1552 (59.3) |  |
|  | (Missing) | * | * | * |  |
| Total days with any organ support | Median (IQR) | 3.0 (1.0 to 6.0) | 2.0 (1.0 to 3.0) | 2.0 (1.0 to 6.0) | <0.0001 |
| Maximum number of organs supported across admission | 0 | 293 (13.7) | 77 (15.8) | 370 (14.1) | <0.0001 |
|  | 1 | 572 (26.8) | 227 (46.6) | 799 (30.5) |  |
|  | 2 | 903 (42.4) | 160 (32.9) | 1063 (40.6) |  |
|  | 3 | 363 (17.0) | 23 (4.7) | 386 (14.7) |  |
|  | (Missing) | * | * | * |  |
| ICU mortality | Survivors | 1268 (59.4) | 391 (80.5) | 1659 (63.3) | <0.0001 |
|  | Non-Survivors | 865 (40.6) | 95 (19.5) | 960 (36.7) |  |
|  | (Missing) | * | * | * |  |
| Hospital mortality | Survivors | 979 (46.5) | 317 (65.5) | 1296 (50.1) | <0.0001 |
|  | Non-Survivors | 1126 (53.5) | 167 (34.5) | 1293 (49.9) |  |
|  | (Missing) | * | * | 31 |  |
| 60-day mortality | Survivors | 991 (46.5) | 306 (63.0) | 1297 (49.5) | <0.0001 |
|  | Non-Survivors | 1142 (53.5) | 180 (37.0) | 1322 (50.5) |  |
|  | (Missing) | * | * | * |  |
| Two-year mortality | Survivors | 604 (29.9) | 184 (40.4) | 788 (31.8) | <0.0001 |
|  | Non-Survivors | 1417 (70.1) | 271 (59.6) | 1688 (68.2) |  |
|  | (Missing) | 112 | 32 | 144 |  |
| Five-year mortality | Survivors | 252 (13.8) | 59 (14.8) | 311 (13.9) | 0.6624 |
|  | Non-Survivors | 1579 (86.2) | 341 (85.2) | 1920 (86.1) |  |
|  | (Missing) | 302 | 87 | 389 |  |
| ICU, intensive-care unit; ALD, alcohol-related liver disease.  * Values <10 suppressed due to disclosure risk.  Range is not reported with median (IQR) due to disclosure risk for values <10. | | | | | |

**Table S16** Subgroup analysis: Length of stay in ICU and hospital for patients with ALD admitted to ICU with and without a GI variceal bleed (n=2,620). Length of stay is stratified according to ICU and hospital survivorship. P-value is calculated using the Mann-Whitney U test or Kruskal-Wallis test for numerical data and χ2-squared test for categorical data. Results are summarized as median (IQR).

| **Outcome** |  | **GI bleeding: No (n=1,268)** | **GI bleeding: Yes (n=391)** | **Total (n=1,659)** | **p-value** |
| --- | --- | --- | --- | --- | --- |
| ICU length of stay (ICU survivors) | Median (IQR) | 4.0 (2.0 to 8.0) | 1.0 (1.0 to 3.0) | 3.0 (1.0 to 7.0) | <0.0001 |

| **Outcome** |  | **GI bleeding: No (n=865)** | **GI bleeding: Yes (n=95)** | **Total (n=960)** | **p-value** |
| --- | --- | --- | --- | --- | --- |
| ICU length of stay (ICU non-survivors) | Median (IQR) | 2.0 (1.0 to 5.0) | 3.0 (1.0 to 6.0) | 2.0 (1.0 to 5.0) | 0.4775 |

| **Outcome** |  | **GI bleeding: No (n=979)** | **GI bleeding: Yes (n=317)** | **Total (n=1,296)** | **p-value** |
| --- | --- | --- | --- | --- | --- |
| Hospital length of stay (hospital survivors) | Median (IQR) | 18.0 (9.0 to 35.0) | 10.0 (6.0 to 18.0) | 16.0 (8.0 to 31.0) | <0.0001 |

| **Outcome** |  | **GI bleeding: No (n=1,126)** | **GI bleeding: Yes (n=167)** | **Total (n=1,293)** | **p-value** |
| --- | --- | --- | --- | --- | --- |
| Hospital length of stay (hospital non-survivors) | Median (IQR) | 3.0 (1.0 to 10.0) | 6.0 (2.0 to 13.0) | 4.0 (1.0 to 10.0) | 0.0012 |
| GI, gastrointestinal; ICU, intensive care unit; IQR, interquartile range.  Range is not reported with median (IQR) due to disclosure risk for values <10. | | | | | |

**Table S17** Readmission within two years (hospital survivors only) following index ICU admission for patients with ALD admitted to ICU with and without a GI variceal bleed (n=2,620). P value was calculated using the χ2-squared test. Results are summarized as number (%).

| **Outcome** |  | **GI bleeding: No (n=979)** | **GI bleeding: Yes (n=317)** | **Total (n=1296)** | **p-value** |
| --- | --- | --- | --- | --- | --- |
| ICU readmission within 2 years | No | 713 (81.7) | 196 (67.8) | 909 (78.2) | <0.0001 |
|  | Yes | 160 (18.3) | 93 (32.2) | 253 (21.8) |  |
|  | (Missing) | 106 | 28 | 134 |  |
| Hospital readmission within 2 years | No | 146 (15.4) | 32 (10.2) | 178 (14.1) | 0.0266 |
|  | Yes | 800 (84.6) | 282 (89.8) | 1082 (85.9) |  |
|  | (Missing) | * | * | 36 |  |
| ICU, intensive care unit; GI, gastrointestinal.  * Values <10 suppressed due to disclosure risk. | | | | | |

**Table S18** Sensitivity analysis: Individual results for the multivariable regression analysis (Figure S5) for factors associated with ICU mortality, restricted to patients with liver disease in ICU (including ALD) (n=5,030). P-values are calculated using the Mann-Whitney U or Kruskal-Wallis test for numerical data and chi-squared test for categorical data. Results are summarized as percentage (%) or odds ratio (CI, p-value).

| **Dependent: ICU mortality** |  | **Survivors** | **Non-Survivors** | **OR (univariable)** | **OR (multivariable)** |
| --- | --- | --- | --- | --- | --- |
| ALD diagnosis on or before index admission | No | 1602 (66.8) | 798 (33.2) | - | - |
|  | Yes | 1664 (63.3) | 964 (36.7) | 1.16 (1.04-1.31, p=0.0109) | 1.40 (1.21-1.61, p<0.0001) |
| Age | 18-44 | 788 (70.5) | 329 (29.5) | - | - |
|  | 45-54 | 835 (66.0) | 430 (34.0) | 1.23 (1.04-1.47, p=0.0178) | 1.18 (0.97-1.44, p=0.0939) |
|  | 55-64 | 873 (63.2) | 508 (36.8) | 1.39 (1.18-1.65, p=0.0001) | 1.28 (1.05-1.55, p=0.0143) |
|  | 65-74 | 542 (59.7) | 366 (40.3) | 1.62 (1.34-1.95, p<0.0001) | 1.71 (1.38-2.12, p<0.0001) |
|  | 75+ | 228 (63.9) | 129 (36.1) | 1.36 (1.05-1.74, p=0.0178) | 1.73 (1.29-2.33, p=0.0003) |
| Sex | Male | 1878 (64.8) | 1019 (35.2) | - | - |
|  | Female | 1388 (65.1) | 743 (34.9) | 0.99 (0.88-1.11, p=0.8210) | 1.00 (0.88-1.15, p=0.9536) |
| SIMD | 1 (most deprived) | 1101 (63.2) | 640 (36.8) | - | - |
|  | 2 | 811 (65.6) | 425 (34.4) | 0.90 (0.77-1.05, p=0.1828) | 0.93 (0.78-1.10, p=0.3936) |
|  | 3 | 546 (63.7) | 311 (36.3) | 0.98 (0.83-1.16, p=0.8147) | 0.94 (0.78-1.14, p=0.5260) |
|  | 4 | 451 (69.4) | 199 (30.6) | 0.76 (0.62-0.92, p=0.0052) | 0.77 (0.62-0.95, p=0.0158) |
|  | 5 (least deprived) | 326 (65.2) | 174 (34.8) | 0.92 (0.74-1.13, p=0.4218) | 0.86 (0.68-1.09, p=0.2088) |
| Total Charlson comorbidities (ex. liver disease) | 0 | 1331 (66.5) | 670 (33.5) | - | - |
|  | 1 | 1024 (63.3) | 593 (36.7) | 1.15 (1.00-1.32, p=0.0454) | 1.13 (0.96-1.32, p=0.1318) |
|  | 2+ | 911 (64.6) | 499 (35.4) | 1.09 (0.94-1.26, p=0.2479) | 1.06 (0.89-1.25, p=0.5382) |
| APACHE ICU admission diagnosis (system) | GI disorder | 1075 (66.9) | 531 (33.1) | - | - |
|  | Cardiovascular disorder | 653 (49.8) | 659 (50.2) | 2.04 (1.76-2.37, p<0.0001) | 1.39 (1.17-1.66, p=0.0002) |
|  | Respiratory disorder | 626 (64.5) | 345 (35.5) | 1.12 (0.94-1.32, p=0.2002) | 1.03 (0.85-1.24, p=0.7662) |
|  | Neurological disorder | 484 (79.2) | 127 (20.8) | 0.53 (0.42-0.66, p<0.0001) | 0.54 (0.42-0.68, p<0.0001) |
|  | Metabolic/renal disorder | 335 (80.0) | 84 (20.0) | 0.51 (0.39-0.66, p<0.0001) | 0.50 (0.37-0.66, p<0.0001) |
|  | Trauma | 85 (89.5) | 10 (10.5) | 0.24 (0.12-0.44, p<0.0001) | 0.37 (0.18-0.71, p=0.0053) |
| Acute Physiology Score (APS) | Mean (SD) | 14.7 (7.8) | 21.0 (10.9) | 1.08 (1.07-1.09, p<0.0001) | 1.05 (1.04-1.06, p<0.0001) |
| Invasive mechanical ventilation (day 1) | No | 1292 (77.2) | 381 (22.8) | - | - |
|  | Yes | 1971 (58.9) | 1378 (41.1) | 2.37 (2.08-2.71, p<0.0001) | 1.56 (1.34-1.83, p<0.0001) |
| Renal support (day 1) | No | 2966 (68.8) | 1343 (31.2) | - | - |
|  | Yes | 297 (41.7) | 416 (58.3) | 3.09 (2.63-3.64, p<0.0001) | 1.71 (1.41-2.07, p<0.0001) |
| Cardiovascular support (day 1) | No | 2020 (80.2) | 500 (19.8) | - | - |
|  | Yes | 1243 (49.7) | 1259 (50.3) | 4.09 (3.61-4.64, p<0.0001) | 2.26 (1.95-2.61, p<0.0001) |
| OR, odds ratio; SIMD, Scottish Index of Multiple Deprivation; GI disorder, gastrointestinal disorder; APACHE, Acute Physiology And Chronic Health Evaluation. | | | | | |

**Table S19** Sensitivity analysis: Individual results for the multivariable regression analysis (figure E6) identifying factors associated with ICU mortality in all patients in ICU excluding patients non-ALD liver disease (n=47,019). P-values are calculated using the Mann-Whitney U or Kruskal-Wallis test for numerical data and chi-squared test for categorical data. Results are summarized as percentage (%) or odds ratio (CI, p-value).

| **Dependent: ICU mortality** |  | **Survivors** | **Non-Survivors** | **OR (univariable)** | **OR (multivariable)** |
| --- | --- | --- | --- | --- | --- |
| ALD diagnosis on or before index admission | No | 34620 (78.1) | 9719 (21.9) | - | - |
|  | Yes | 1664 (63.3) | 964 (36.7) | 2.06 (1.90-2.24, p<0.0001) | 2.15 (1.95-2.37, p<0.0001) |
| Age | 18-44 | 10484 (88.6) | 1343 (11.4) | - | - |
|  | 45-54 | 6345 (80.9) | 1495 (19.1) | 1.84 (1.70-1.99, p<0.0001) | 1.26 (1.16-1.38, p<0.0001) |
|  | 55-64 | 7032 (75.1) | 2329 (24.9) | 2.59 (2.40-2.78, p<0.0001) | 1.58 (1.45-1.72, p<0.0001) |
|  | 65-74 | 7190 (70.2) | 3055 (29.8) | 3.32 (3.09-3.56, p<0.0001) | 2.11 (1.94-2.29, p<0.0001) |
|  | 75+ | 5233 (68.0) | 2461 (32.0) | 3.67 (3.41-3.96, p<0.0001) | 2.83 (2.59-3.09, p<0.0001) |
| Sex | Male | 20655 (77.1) | 6149 (22.9) | - | - |
|  | Female | 15428 (77.4) | 4502 (22.6) | 0.98 (0.94-1.02, p=0.3703) | 1.02 (0.97-1.07, p=0.5048) |
| SIMD | 1 (most deprived) | 10469 (77.6) | 3018 (22.4) | - | - |
|  | 2 | 8877 (77.7) | 2545 (22.3) | 0.99 (0.94-1.06, p=0.8568) | 0.96 (0.89-1.02, p=0.1899) |
|  | 3 | 6779 (77.5) | 1970 (22.5) | 1.01 (0.95-1.08, p=0.8072) | 0.92 (0.85-0.99, p=0.0206) |
|  | 4 | 5226 (76.4) | 1617 (23.6) | 1.07 (1.00-1.15, p=0.0442) | 0.95 (0.88-1.03, p=0.2099) |
|  | 5 (least deprived) | 4141 (74.9) | 1389 (25.1) | 1.16 (1.08-1.25, p<0.0001) | 1.04 (0.96-1.13, p=0.3520) |
| Total Charlson comorbidities (ex. liver disease) | 0 | 15146 (84.5) | 2771 (15.5) | - | - |
|  | 1 | 11424 (74.9) | 3825 (25.1) | 1.83 (1.73-1.93, p<0.0001) | 1.44 (1.35-1.53, p<0.0001) |
|  | 2+ | 9513 (70.1) | 4055 (29.9) | 2.33 (2.21-2.46, p<0.0001) | 1.53 (1.43-1.63, p<0.0001) |
| APACHE ICU admission diagnosis (system) | GI disorder | 3127 (76.1) | 981 (23.9) | - | - |
|  | Cardiovascular disorder | 9126 (65.2) | 4875 (34.8) | 1.70 (1.57-1.84, p<0.0001) | 1.40 (1.28-1.53, p<0.0001) |
|  | Respiratory disorder | 9454 (78.8) | 2549 (21.2) | 0.86 (0.79-0.94, p=0.0004) | 0.95 (0.86-1.05, p=0.3035) |
|  | Neurological disorder | 5763 (78.0) | 1625 (22.0) | 0.90 (0.82-0.98, p=0.0207) | 1.07 (0.96-1.19, p=0.2145) |
|  | Metabolic/renal disorder | 5801 (95.1) | 300 (4.9) | 0.16 (0.14-0.19, p<0.0001) | 0.23 (0.20-0.27, p<0.0001) |
|  | Trauma | 2830 (90.0) | 316 (10.0) | 0.36 (0.31-0.41, p<0.0001) | 0.75 (0.64-0.87, p=0.0002) |
| Acute Physiology Score (APS) | Mean (SD) | 12.9 (7.6) | 18.8 (10.8) | 1.08 (1.08-1.09, p<0.0001) | 1.05 (1.05-1.05, p<0.0001) |
| Invasive mechanical ventilation (day 1) | No | 15879 (88.9) | 1985 (11.1) | - | - |
|  | Yes | 20386 (70.1) | 8687 (29.9) | 3.41 (3.23-3.60, p<0.0001) | 2.35 (2.21-2.50, p<0.0001) |
| Renal support (day 1) | No | 34296 (78.6) | 9354 (21.4) | - | - |
|  | Yes | 1969 (59.9) | 1318 (40.1) | 2.45 (2.28-2.64, p<0.0001) | 1.38 (1.26-1.51, p<0.0001) |
| Cardiovascular support (day 1) | No | 23939 (87.3) | 3492 (12.7) | - | - |
|  | Yes | 12326 (63.2) | 7180 (36.8) | 3.99 (3.81-4.18, p<0.0001) | 1.93 (1.83-2.04, p<0.0001) |
| OR, odds ratio; SIMD, Scottish Index of Multiple Deprivation; GI disorder, gastrointestinal disorder; APACHE, Acute Physiology And Chronic Health Evaluation. | | | | | |
